# The Arch from the Stones: Understanding Protein Folding Energy Landscapes via Bioinspired Collective Variables

**DOI:** 10.1021/acs.jpclett.5c02079

**Published:** 2025-09-08

**Authors:** Valerio Rizzi, Margaux Héritier, Nicola Piasentin, Simone Aureli, Francesco Luigi Gervasio

**Affiliations:** † School of Pharmaceutical Sciences, University of Geneva, Rue Michel-Servet 1, CH-1206 Geneva, CH, Switzerland; ‡ Institute of Pharmaceutical Sciences of Western Switzerland, 27212 University of Geneva, CH-1206, Geneva, CH, Switzerland; ¶ Swiss Bioinformatics Institute, University of Geneva, CH-1206, Geneva, CH, Switzerland; ∥ Department of Chemistry, 4919 University College London, London, WC1E 6BT, United Kingdom

## Abstract

Protein folding remains a formidable challenge despite significant advances, particularly in sequence-to-structure prediction. Accurately capturing thermodynamics and intermediates via simulations demands overcoming time scale limitations, making effective collective variable (CV) design for enhanced sampling crucial. Here, we introduce a strategy to automatically construct complementary, bioinspired CVs. These uniquely capture local hydrogen bonding—explicitly distinguishing protein–protein from protein–water interactions—and side-chain packing, taking into account both native and non-native contacts to enhance state resolution. Using these CVs in combination with advanced enhanced sampling methods, we simulate the folding of Chignolin and TRP-cage, validating our approach against extensive unbiased simulations. Our results accurately resolve complex free-energy landscapes, reveal critical intermediates such as the dry molten globule, and demonstrate agreement with reference data. This interpretable and portable strategy underscores the critical role of microscopic details in protein folding, opening up a promising avenue for studying larger and more-complex biomolecular systems.

## Graphical abstract

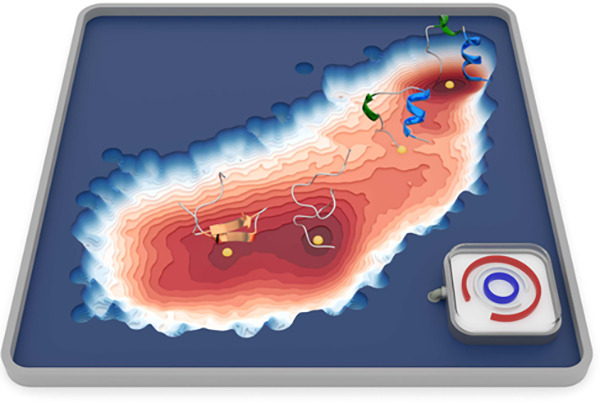

*Marco Polo describes a bridge, stone by stone. -But which stone supports the bridge? - asks Kublai Khan. - The bridge is not supported by this or that stone, - answers Marco, - but by the arch that they form. Kublai Khan keeps silent, thinking. Then he adds: -Why are you talking about the stones then? I am interested only in the arch. Polo replies: - Without stones there is no arch*.^1^ The bridge described by Marco Polo to Kublai Khan in Italo Calvino’s Invisible Cities bears a remarkable resemblance to a folded protein with its global shape emerging naturally when all its interactions, the bridge’s stones, are in one place and no other.

Protein folding is a fundamental problem in biochemistry, with the structure–function paradigm being a cornerstone of biology and drug discovery.^2–6^ Many decades have passed since the discovery that amino acid sequences determine how proteins fold into three-dimensional shapes,^7^ and significant progress has been made in the field until the recent recognition of the 2024 Nobel Prize in Chemistry awarded to the developers of Alpha Fold.^8^ While the advent of Alpha Fold has made a significant leap forward in solving the problem of associating a structure to a sequence,^8^ several crucial aspects of protein folding are still largely unsolved and subject to intense research efforts,^9^ namely, the microscopic details of how a protein folds,^10,11^ the role of folding intermediates,^12–15^ and the systematic determination of thermodynamic and kinetic folding properties.^16–19^

If all protein configurations were equally accessible and the folding probability landscape was flat like a golf course, the famous Levinthal paradox would apply,^20,21^ making the folding time unfathomably long. On the contrary, at room temperature, energetic considerations discourage visiting an exceedingly large number of configurations, such as those where hydrogen bonds are unsatisfied.^5,6,22^ The folding probability landscape must resemble a rugged funnel that is largest in the entropic unfolded basin and becomes narrower and narrower as one approaches the folded state.^23–26^ The metastable intermediates that populate the folding landscape can differ from each other by the smallest of details, such as the orientation of a side chain^22,27^ or the presence/absence of a single water molecule in a key position.^28,29^ Progress on this landscape is made by incremental stochastic steps that bring one closer and closer to the global free-energy minimum, while crossing a myriad of small kinetic barriers.^5^

Computer simulations are a powerful tool to shed light on the protein folding problem. In this respect, simplified models such as Go̅ models have greatly contributed to clarifying the general features of the protein folding landscape.^30^ However, by construction, these models forego a detailed atomistic description. Atomistic molecular dynamics (MD) simulations would be well-suited to the task, but are limited by the time scale problem.^31^ Protein folding events tend to occur over hundreds of microseconds, and notwithstanding the continuous progress of computer hardware, reaching such long time scales remains a demanding task even today. Running long MD simulations on Anton,^32,33^ a specialized computer for MD, produced groundbreaking results, generating folding trajectories of small proteins and predicting kinetic and thermodynamic properties close to the experimental ones.^16,34–37^ However, the inaccessibility of Anton to the wider academic community and the cost of designing specialized hardware limits the practical use of such an approach.

Enhanced sampling algorithms provide a valid alternative to running extremely long MD simulations on specialized hardware. In this respect, methods based on replica exchange techniques^38–42^ and on collective variables (CV) have been used on protein folding with some success.^19,43,44^ However, even collective variable-based enhanced sampling methods such as Metadynamics^45,46^ and On-the-fly Probability Enhanced Sampling (OPES)^47–49^ are limited by the quality of the chosen CVs that should properly encode the phenomenon of interest.^50–52^ Recently, we have developed a hybrid sampling scheme called OneOPES^53^ that, by exploiting the power of OPES Explore,^49^ agnostic techniques such as OPES MultiThermal^48^ and replica exchange,^54^ makes the requirement to develop optimal CVs a less severe but still very valuable endeavor.

Good-quality CVs must first be able to distinguish the end states, the unfolded and folded state, and then capture possible intermediates along the folding path and encode as many as possible slow degrees of freedom (DOFs) that yield the folding/unfolding kinetic bottlenecks. A number of folding CVs have been developed over the years, from the ones that capture global properties such as the root-mean-square deviation (RMSD) away from a state, the radius of gyration,^43^ or the number of turns of alpha helices^55^ to CVs that combine a number of local features such as interatomic distances or dihedrals according to different criteria.^44,56–64^

One of the main limitations shared by existing folding CVs is degeneracy, i.e., the fact that certain CV values are associated with multiple relevant states. This makes it extremely difficult for a CV to accurately resolve well the multitude of metastable states relevant to folding, especially in close proximity to the native folded state. Accelerating degenerate CVs slows down recrossings, leads to hysteresis, and hampers convergence. CVs that encode global properties are intrinsically affected by this problem as they typically cannot resolve the microscopic interactions that characterize the folded basin. For example, CVs that exclusively focus on the protein’s backbone such as interatomic distances between Cαs or dihedrals do not resolve the reciprocal position of the side chains and do not have an explicit focus on capturing hydrogen bonds. The degeneracy problem increases in severity with system size: attempts to use CV-based enhanced sampling to converge the folding free energy on proteins larger than the standard 10 residue Chignolin mini-protein,^65,66^ such as the 20-residue TRP-cage mini-protein,^67^ are few^68^ and less successful.^59^

In this context, the elephant in the room may be the role of the solvent molecules, which account for the vast majority of the atoms in a simulation box. Many interactions play a role in the folding of globular proteins, among which electrostatic interactions and steric hindrance, but hydrophobicity has emerged possibly as the leading factor.^69^ The egress of water molecules from the protein hydrophobic core and the replacement of solvent-mediated hydrogen bonds with direct protein–protein hydrogen bonds are key steps in which water plays an essential role. Accelerating the dynamics of water molecules at relevant positions is an idea that is gaining traction,^70–72^ and we have recently proposed specialized water CVs in different scenarios, such as ligand binding,^73–77^ ion transport,^78^ and local conformational changes.^79,80^

In this work, we develop a bottom-up strategy that builds two complementary CVs, starting from the selection of bioinspired features. One CV focuses on capturing individual high-energy microscopic interactions (hydrogen bonds that identify and distinguish protein–protein from protein–water bonds, while also encoding angular information). The other captures mesoscale contacts between side-chains, encoding compactness. The features are automatically collected from short unbiased end-state trajectories and filtered using a Linear Discriminant Analysis (LDA)-like criterion.^81^ For both CVs, a sharp distinction between the true folded state and defective metastable states is achieved by summing native contacts and explicitly subtracting non-native contacts. Finally, the two CVs are intuitively merged as a simple linear combination of features with unitary coefficients.

We use such CVs to investigate the folding free energy of two well-known mini-proteins from the seminal paper of Lindorff-Larsen et al.:^16^ Chignolin^65,66^ and TRP-cage.^67^ The references for all simulations are self-generated long unbiased trajectories of 300 μs for Chignolin and 200 μs for TRP-cage, which are made available for future reference and CV development. The enhanced sampling simulations are run in quintuplicate to ensure reproducible results. For Chignolin, both single-replica OPES and OneOPES simulations converge to analogous results, with OneOPES being faster and more robust. For TRP-cage, OneOPES calculations are in good agreement with the reference. We analyze the resulting folding landscape, focusing on the metastable intermediate states leading to the native state, such as the elusive dry molten globule (DMG).^12,13,15,82^

The convergence speed and quality across the set of examples, combined with the intuitive and portable nature of the CVs, support our claim that the construction of highly optimized microscopic features represents the crucial element to be accelerated in folding simulations, confirming the paradigm that folded structures emerge naturally from the fulfillment of microscopic contacts. Our automated strategy is well-suited for combination with modern machine learning strategies^83–85^ and can pave the way for in-depth studies of larger and more-complex proteins.

## A Bottom-up Strategy for Folding Featurization

An effective approach to designing CVs involves initially observing and defining a number of features of the system under investigation and then combining these features into CVs at a later stage. To accelerate the process of interest in enhanced sampling, CVs and features must first be able to distinguish states and follow the process through the phase space. Ideally, they should align themselves with the lowest free-energy path and approximate the committor.^62,63,86^

While a rather large number of approaches for combining features exists, the features’ search and optimization are often neglected, despite being a more fundamental aspect than the combination stage. In fact, an incomplete or suboptimal choice of features that ignores crucial degrees of freedom has no chance of producing an optimal CV, even when coalesced with the most sophisticated combination techniques.^52^

A common choice of features is the set of all interatomic distances or contacts between heavy atoms.^44,64,87–90^ This is often an excellent option, especially in low-density gas phase systems, as it captures a large amount of information and can distinguish well microstates while respecting translational, rotational, and permutational symmetries. However, in protein folding, this choice can be suboptimal. For instance, the number of distances grows quadratically with the number of atoms, requiring a preliminary selection of a subset of atoms for manageability. The distances between Cαs are a common choice, but they do not encode any information about the side-chain movements and do not explicitly detect native and non-native contacts. Such information is essential for resolving the intermediate metastable states, or kinetic traps, that populate the rugged folding landscape and requires a level of microscopic detail that Cα distances alone cannot furnish.

Finally, and perhaps most importantly, any set of protein–protein distances neglects the role of the solvent. The solvent, frequently considered less important, holds a significance at least equivalent to that of the protein atoms, especially in satisfying hydrogen bonding. In fact, the energy contribution of a *single* hydrogen bond to the total potential energy lies in the range of a few kcal/mol, which is comparable to the whole folding free-energy difference of many small proteins. Consequently, the total number of hydrogen bonds tends to be a highly conserved quantity throughout a folding/unfolding process.^5,22,91^ Breaking an intraprotein hydrogen bond simply by stretching the corresponding protein–protein distance risks crossing high-energy, improbable states, as no alternative hydrogen bond is promptly available for replacement. Instead, simultaneously driving a water molecule into the correct position to replace the cleaved hydrogen bond helps to reduce the free-energy cost of crossing the barrier, thereby better approximating the minimum free-energy path.

Because of these considerations, in this paper, we propose a bottom-up strategy to build folding CVs starting from picking and optimizing two key sets of features (see Figure 1):

- the formation and cleavage of hydrogen bonds $\left(F_{i}^{HB}\right)$ ;
- the relative packing of the side chains $\left(F_{i}^{SC}\right)$ .

Both are crucial to ensure a comprehensive description of secondary structures (α helices and β sheets) and disordered portions. In the final CVs, features that capture native contacts that exist in the folded basin $\left(F_{i}^{HB/SC,F}\right)$ are summed with coefficient 1, while those that capture contacts from the unfolded basin $\left(F_{i}^{\mathrm{HB/SC,U}}\right)$ have coefficient −1. The two resulting CVs are simply

1 $$s^{HB}=\underset{i}{\sum }F_{i}^{HB,F}-F_{i}^{HB,U}$$

2 $$s^{SC}=\underset{i}{\sum }F_{i}^{SC,F}-F_{i}^{SC,U}$$

Individual features $\left(F_{i}^{HB/SC,F/U}\right)$ act as a switch, going from ∼1 when a native contact is present to ∼0 when no contact is present, and toward negative values in the case of a non-native contact. In the , we detail the construction of both sets, their filtering process, and their combination into CVs. Our workflow requires only two short unbiased trajectories, indicatively 100 ns long, one in the folded basin and one in the unfolded basin, and it is fully automated and available as a Python script. The script output consists of PLUMED^92^ files, ready for either trajectory post-processing or enhanced sampling simulations.

## Chignolin

At first, we simulate the folding of a well-known system that has been widely used in method testing over the years: the double mutant CLN025 of the Chignolin miniprotein.^65,66^ Our simulation input parameters match, when possible, the ones from ref 16 (see the for more details), including a simulation temperature of 340 K. We use, as a reference, a self-generated set of 3 unbiased trajectories, each 100 μs long (see ) containing a total of 76 back-and-forth folding transitions. We observe a mean unfolding time of 3.5 μs and a mean folding time of 0.3 μs. To estimate these quantities, we follow the dual-cutoff approach from ref 16 with a time window of 10 ns. We use the RMSD over the Cα atoms as a discriminant, with cutoffs for the folded state of 1.5 and 2.5 Å for the unfolded state.

We use these unbiased simulations to calculate our reference folding free energy Δ*F* = −1.66 ± 0.16 kcal mol^–1^ and observe a slight shift, compared to the reported value of −0.9 kcal mol^–1^.^16^ We believe this to be due to subtle differences in the implementation of the force field in different MD codes. To investigate where the energy shift emerges, we evaluate the enthalpy difference Δ*H* by partitioning the total energy of our unbiased NVT trajectories into folded and unfolded basins (see ). Our resulting Δ*H* = 7.2 ± 0.2 kcal mol^–1^ is shifted with respect to the value reported in ref 16 Δ*H* = 6.1 ± 0.1 kcal mol^–1^ by a similar amount as the folding free energy Δ*F*, giving credit to our hypothesis of slight force field discrepancies.

To test our CVs in the context of enhanced sampling, we first perform single replica simulations of the system biasing *s* ^HB^ and *s* ^SC^ with OPES^47^ (see Figure 2, as well as ). In this and in the following examples, we always estimate the free energy and its error as the mean and the standard deviation of five independent simulations (see the for more details). These single replica simulations already represent a rather stringent CV test, where suboptimal CVs are known to display hysteresis and evident discrepancies in the estimated Δ*F* between independent simulations. Instead, our Δ*F*(*t*) estimate is consistently aligned in all replicas with the expected value after about 400 ns (see Figure 2a) and remains rather constant throughout the rest of the simulations, giving a final estimate of Δ*F* = −1.81 ± 0.20 kcal mol^–1^. The FES profile along the commonly used RMSD over the Cα atoms is in good agreement with the unbiased reference (see Figure 2d).

**Figure 1:**
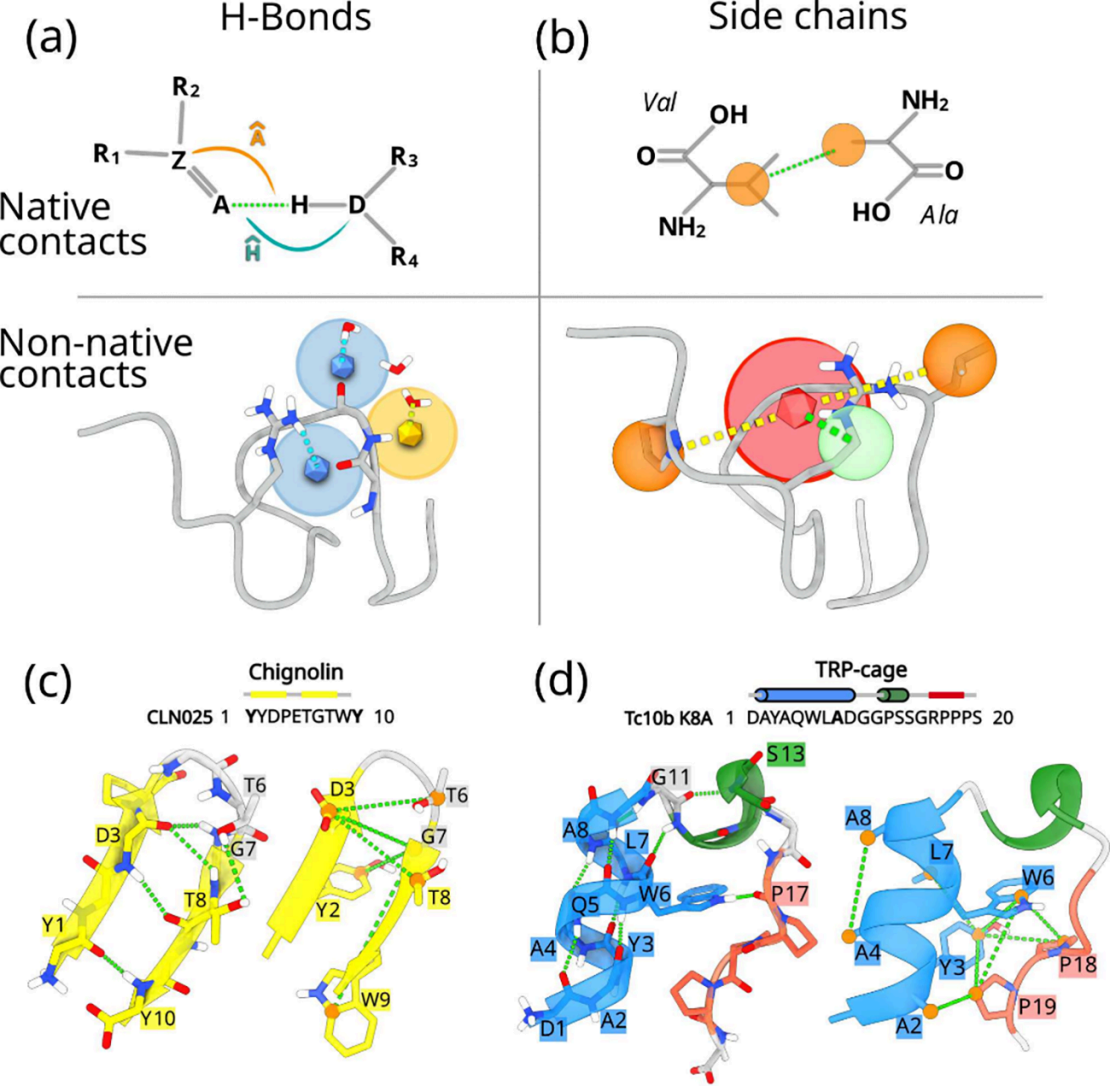
Feature design and systems studied. (a) Illustrative definition of a hydrogen bond feature considering the bond angular components for native contacts and the protein–protein and protein–water interactions for non-native contacts. (b) Illustrative definition of the side chain feature with both a native and a non-native interaction between centers of mass of side chains. (c, d) Sequence and 3D native structures of Chignolin and TRP-cage, respectively. α-helices are represented in blue, 3_10_-helices in green, β-helices in yellow, and random coils in gray. The green dashed lines highlight the most relevant hydrogen-bond contacts (left) and the most relevant side-chain contacts (right).

To highlight the crucial role of the inclusion of non-native contacts in the CVs, we build abridged CVs where each individual feature $F_{i}^{HB/SC,F/U}$ retains only the description of native contacts and all non-native contacts are instead switched off (see the for more details). The resulting Δ*F* and FES along the RMSD over the Cα atoms are clearly shifted compared to the reference (see Figures 2b and 2e) with a slow tendency to approach it in the long time limit. This behavior is a typical indicator of CVs missing important degrees of freedom and stresses the importance of capturing and including non-native contacts. In , we show analogous results where we selectively turned off non-native interactions with water and with protein atoms, respectively. We observe the worst results when excluding non-native protein contacts that, in this case, prove to be the most essential to retain.

We then perform OneOPES simulations with our standard CVs and observe a faster convergence and an even stronger agreement with the reference (see Figures 2c and 2f, as well as ), as well as a more consistent behavior among the independent simulations than in the single replica case. This is expected, as the use of explicit replica exchange and the bias over additional CVs helps to accelerate degrees of freedom that are not directly sped up by the main CVs. Our final folding free-energy estimate is Δ*F* = −1.64 ± 0.15 kcal mol^–1^ and compares well with the reference (see Table 1). We note that a direct comparison between single replica and multireplica simulations in terms of computational cost is not straightforward, as the parallelism granted by GPU computing greatly helps in reducing the cost of running multireplica systems. All OneOPES free energy estimations are calculated over replica 0, as detailed in ref 53, while there is a total of 8 replicas per independent simulation.

**Table 1:** Free Energies of Folding Δ*F* of the Reference Unbiased Systems and the OneOPES Simulations

	Δ*F* (kcal mol^–1^)	
	OneOPES	reference
Chignolin	–1.64 ± 0.15	–1.66 ± 0.16
TRP-cage	0.91 ± 0.16	0.86 ± 0.27

## TRP-Cage

We now apply our method to study TRP-cage, a more complex mini-protein that has been the focus of a number of experimental^38,67,93,94^ and computational studies.^19,34,37,56,59,68,95^ We study the TC10b K8A single-point mutant that is featured in the seminal work by Shaw et al.,^34^ and we setup the system to match the one from ref 37 (see the SI for more details).

TRP-cage contains 20 residues and features three distinct secondary structure elements: an α-helix spanning residues 1–9, a 3_10_ helix from residue 11 to residue 13, and a polyproline II helix between residues 17–19 (see Figure 1d). These elements assemble into a compact tertiary structure that encapsulates a central tryptophan residue (W6), forming a primitive hydrophobic core in conjunction with residues Y3, G11, P18, and P19. Its richer topology, compared to Chignolin, makes TRP-cage a challenging and informative benchmark for folding simulations. Furthermore, the folding mechanism of TRP-cage can be regarded as a surrogate for the folding of larger proteins, as the rise of its well-defined hydrophobic core entails a hydrophobic collapse and the formation of molten globule intermediate states.^96^

**Figure 2:**
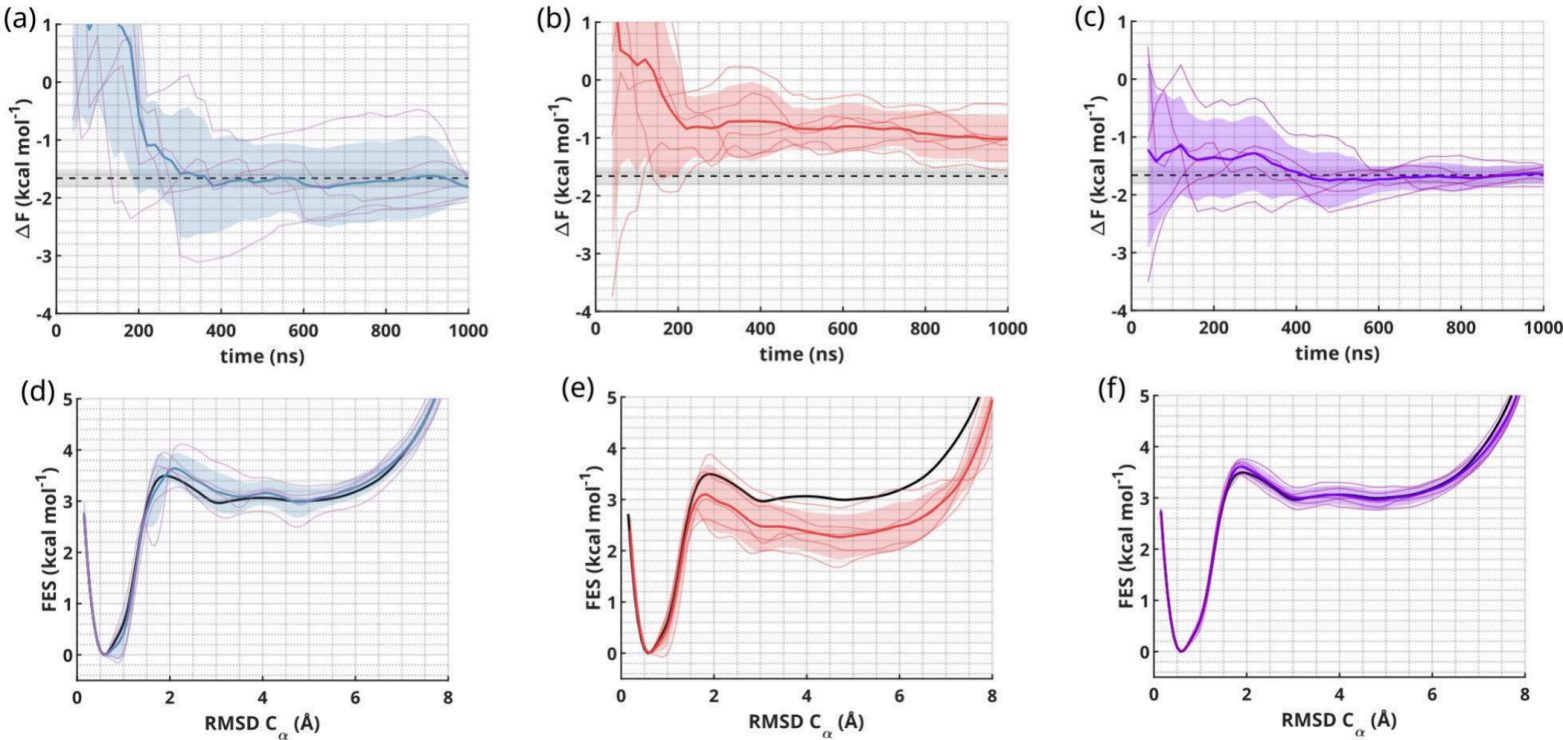
Chignolin folding results. (a–c) Chignolin free-energy difference Δ*F* between the folded and unfolded states over time from five independent OPES trajectories (panel (a)), OPES trajectories with abridged CVs (panel (b)), and OneOPES trajectories (panel (c)). The reference value is displayed as a black dashed line, the trajectories’ average value in solid color, blue, red and purple, respectively, and the standard deviation in semitransparency. (d–f) 1D FES as a function of the RMSD over the Cα atoms for the OPES simulations (panel (d)), OPES simulations with abridged CVs (panel (e)) and the OneOPES simulations (panel (f)). The solid black line shows the reference FES; the solid color lines (blue, red, and purple, respectively) report the average of the five replicas, and the semitransparency indicates their standard deviation.

We use as a reference an extensive self-generated unbiased simulation of 200 μs run at a temperature of 320 K, in analogy with ref 37 (see ). This trajectory contains a total of 26 back-and-forth folding transitions. We observe a mean unfolding time of 1.5 μs and a mean folding time of 6.2 μs. To estimate these quantities, we follow the dual-cutoff approach from ref 16 over the RMSD over the Cα atoms with a time window of 10 ns and cutoffs for the folded state of 4.0 Å and of 6.0 Å for the unfolded state.

From this trajectory, we estimate the folding free energy to be Δ*F* = 0.86 ± 0.27 kcal mol^–1^. After building the *s* ^HB^ and *s* ^SC^ CVs, we run five independent OneOPES simulations (see the for details) and we compare their outcome with the unbiased reference. The folding free energy from the OneOPES simulations, in Figure 3a, displays remarkable agreement with the reference from 500 ns onward. The resulting FES projected on the RMSD over the Cα atoms, in Figure 3b, matches the reference well, with the error peaking in the transition state region, as seen previously with OneOPES applied on other systems.^53^

**Figure 3:**
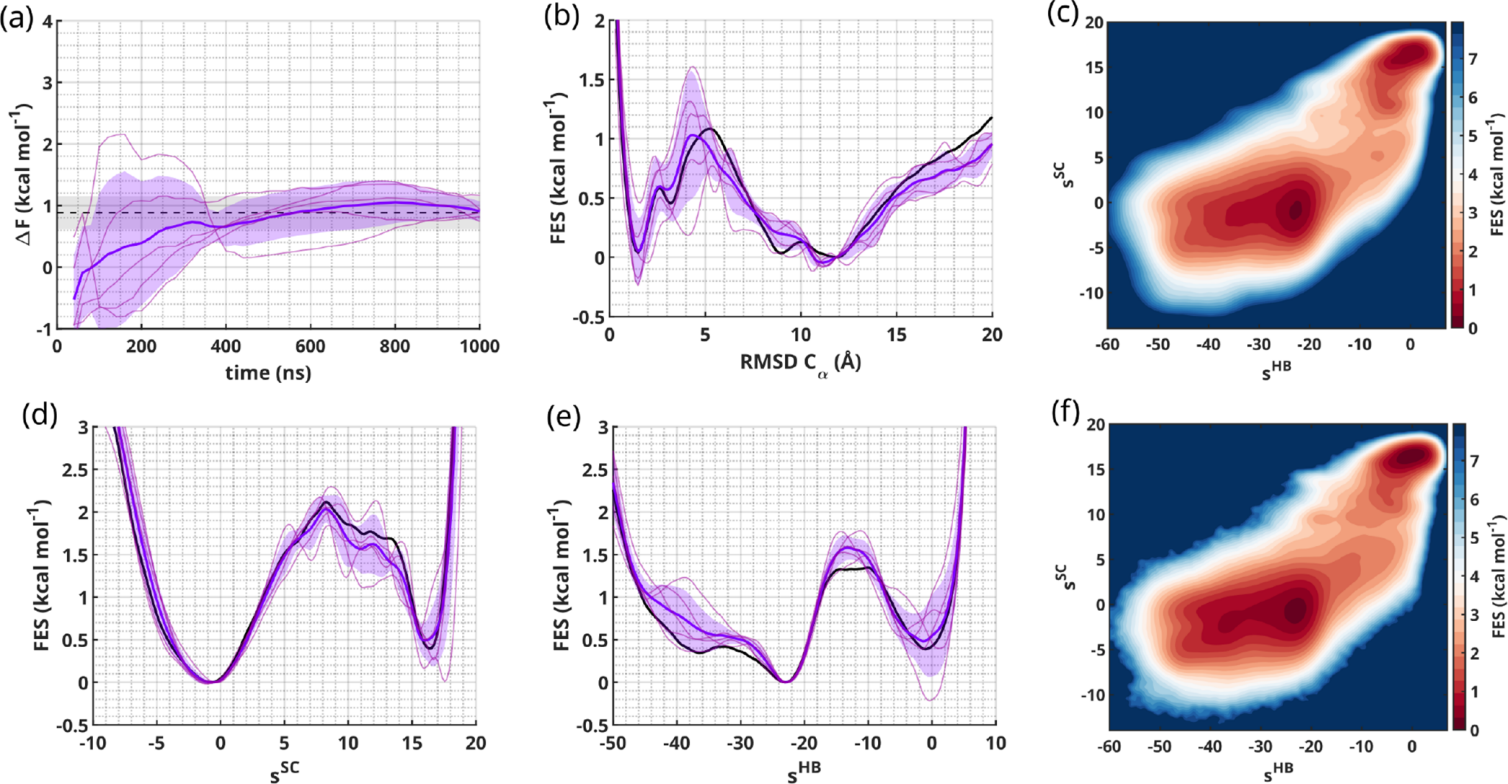
TRP-cage folding results. (a) TRP-cage free-energy difference between the folded and unfolded states over time from five independent OneOPES trajectories. The reference value is displayed as a black dashed line, the trajectories’ average value in solid purple and the standard deviation in semitransparency. (b) 1D FES as a function of the RMSD over the Cα atoms with the reference in solid black, the OneOPES average in solid purple and the standard deviation in semitransparency. (c, f) 2D FES over *s* ^HB^ and *s* ^SC^, with the average value over the OneOPES independent trajectories (panel (c)) and the reference (panel (f)). (d, e) 1D FES as a function of *s* ^SC^ (panel (d)) and *s* ^HB^ (panel (e)), with an analogous color scheme as the one described in panel (b).

The two-dimensional (2D) FES projected on the *s* ^HB^ and *s* ^SC^ CVs, in Figure 3c, closely matches the one built from the reference in Figure 3f and reveals important topological features that deserve a more in-depth analysis. The two main minima corresponding to the unfolded (*s* ^HB^ ≈ −25, *s* ^SC^ ≈ −2) and folded basins (*s* ^HB^ ≈ 0, *s* ^SC^ ≈ 18) are surrounded by plateau-like regions that contain a number of other metastable states. The reweighting of the 2D FES along its one-dimensional components, reported in Figures 3d and 3e, reveals that, while the projection over *s* ^SC^ is dominated by the two main minima, the one over *s* ^HB^ shows an unfolded basin made up of two components.

To probe the source of such bifurcation and further investigate the nature of metastable states, we extract configurations belonging to different regions of the 2D FES and analyze their secondary structure content. As shown in Figure 4, we single out six population ensembles, respectively located in

- the unfolded basin, state U and M (standing for unfolded and misfolded states);
- the transition state region, state WMG (standing for wet molten globule); and
- the folded basin, states F, F2, and DMG (standing for folded and dry molten globule states).

**Figure 4:**
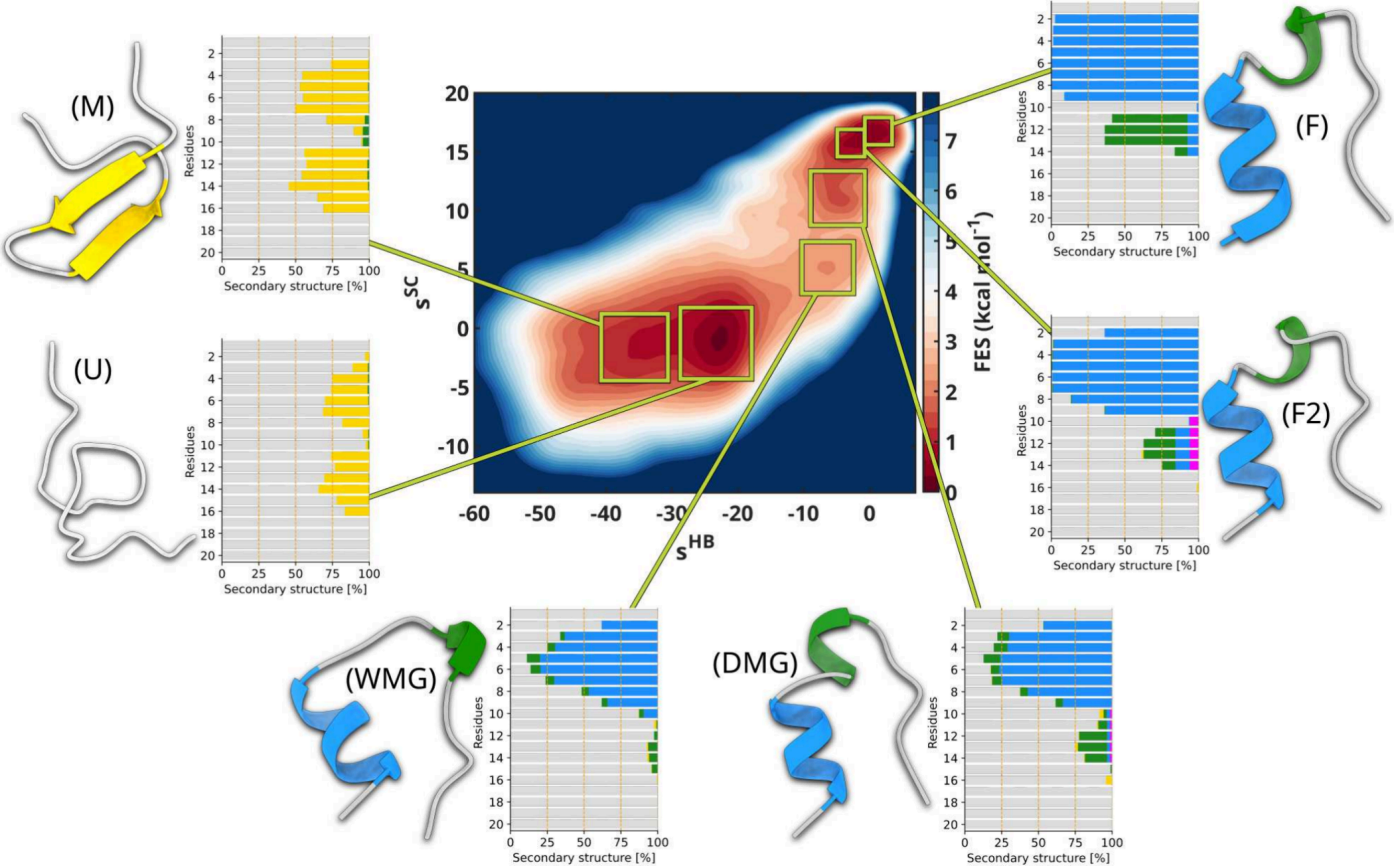
Trp-Cage folding landscape analysis. Six conformational ensembles (M, U, WMG, DMG, F2, and F) extracted from the corresponding regions of the OneOPES TRP-cage 2D FES highlighted by green squares. For each ensemble, on the left, we present a representative 3D structure and on the right a histogram summarizing the secondary structure frequency for each amino acid. α-helices are colored in blue, 3_10_-helices are colored in green, π-helices in magenta, β-helices in yellow, and random coils in gray.

State U is located in the region – 30 ≲ *s* ^HB^ ≲ −20, −4 ≲ *s* ^SC^ ≲ 2 and corresponds to a fully unfolded ensemble of states lacking stable secondary structure, though retaining a modest degree of local order in the regions where the α-helices could form. In contrast, and perhaps surprisingly, state M, located in the interval −40 ≲ *s* ^HB^ ≲ −30 and in a similar *s* ^SC^ range as the above states U, presents a misfolded and partially folded motif, a β-turn-β conformation. To further assess the differences between U and M, we analyze the per-residue solvent-accessible surface area (SASA) and contact frequencies (see ). While the SASA values for the two states are comparable, state M clearly displays contact patterns characteristic of β-hairpins, with increased interactions between distal residues.

The capability of *s* ^HB^ to distinguish U from M is not trivial and is intrinsic to its construction, where non-native contacts are subtracted from the native contacts. While disordered structures contribute almost nothing to *s* ^HB^, misfolded ones contribute negatively, and the more structured they are, the more negative their contribution. The capacity to place misfolded structures on one end of the CV value range is a key quality for a good CV to be used in enhanced sampling as it contributes to clean up the intermediate region from dead ends and kinetic traps. If one looks at a standard CV such as the RMSD over the Cα atoms (see ), its value in state M is indeed mixed with the value of intermediate states that appear in the transition state region. When biased, such CVs might manage to lead the system from U to the transition region but would probably be trapped in M for a long time. Further progress toward state F would be hampered by the necessity to first go backward to the disordered state U before being able to move forward to F. The pitfalls of using such problematic CVs in the context of a simple but pathological 2D potential are well-illustrated in the work by Invernizzi and Parrinello.^49^

In the intermediate region of the free-energy surface −12 ≲ *s* ^HB^ ≲ −2, 4 ≲ *s* ^SC^ ≲ 8, we identify a conformational ensemble that we associate with a wet molten globule (WMG). Structural analysis reveals the emergence of an α-helix spanning residues 3–8, supported by increased contact frequencies within this segment. Notably, the dominant interactions involve Y3, W6, and L7, likely marking the early nucleation site of the hydrophobic core (see ). Despite this local ordering, the per-residue SASA remains high and comparable to that of U and M, indicating that the WMG is still fully solvated.

In the folded region, we identify three states F (folded), F2 (misfolded), and DMG (dry molten globule) that are nearly indistinguishable when characterized using coarse collective variables such as RMSD or AlphaRMSD (see ). In contrast, the atomistic detail encoded in *s* ^HB^ and *s* ^SC^ can resolve the subtle structural differences among them. While states F and F2 feature a well-formed α-helix spanning residues 2–9, state DMG presents a slightly less-ordered helical region that resembles the one present in WMG. The main difference between WMG and DMG lies in per-residue SASA values that significantly drop, especially for W6, G11, and P19, i.e., the key residues involved in hydrophobic core formation. Their decreasing solvent exposure indicates water expulsion and core consolidation, which ultimately lead to the formation and the stabilization of the native-like fold. The SASA values further decrease from DMG to F2, reflecting increasing compactness.

F and F2 closely resemble each other when looking at SASA values but differ locally in the secondary structure. Only state F exhibits persistent folding of the helix’s N- and C-terminal ends, as is expected in a native folded structure. In the region encompassing residues 10–14, F2 shows diverse helical content, including transient 3_10_ and π-helix features, whereas F keeps a stable 3_10_ helix. In , we repeat the same analysis over the same metastable states on the reference unbiased trajectory. The secondary structure content of each basin is largely in agreement with that found in the OneOPES trajectory.

Our results on TRP-cage highlight the power of the *s* ^HB^ and *s* ^SC^ CVs to deliver both accurate thermodynamic estimates of the folding free energy and high-resolution structural information. By capturing subtle structural and hydration differences, our approach is able to accelerate the relevant slow degrees of freedom and offers valuable insights on the ensemble of states along the folding path.

## Conclusions

In this work, we introduce a bottom-up strategy to design physically interpretable CVs capable of grasping the intricate details of protein folding energy landscapes, at both the microscopic and mesoscale level.

A key strength of our approach lies in the complementary nature of the two collective variables. *s* ^HB^ provides a comprehensive atomistic description of hydrogen bonding, capturing secondary structures at a high resolution and efficiently promoting their formation and dissolution in enhanced sampling. Conversely, *s* ^SC^ captures side-chain packing at a coarser scale level, effectively describing and driving hydrophobic collapse and tertiary structure formation. Together, these CVs bridge the gap between microscopic and mesoscale, enabling enhanced sampling simulations to reversibly sample the conformational space until convergence. This synergy proves to be particularly powerful in detecting intermediate and near-native states that might otherwise be overlooked by using traditional CVs alone.

We integrate these CVs in the OneOPES enhanced sampling framework and investigate the folding mechanisms of two mini-proteins: Chignolin and TRP-cage. Our approach yields free-energy landscapes in strong agreement with extensive unbiased simulations. These results underscore the robustness and accuracy of our CVs in quantifying folding thermodynamics. In the case of TRP-cage, we dissect the structural features of relevant basins, resolving and characterizing unfolded, intermediate, and folded states. Our analysis reveals distinct folding routes and highlights the role of hydrophobic core formation in native structure stabilization.

The success of our automated strategy in generating these high-resolution CVs from short, unbiased end-state trajectories is promising. While previous CV-based enhanced sampling studies have often struggled with proteins larger than Chignolin, our results with TRP-cage demonstrate the potential of our approach for more-challenging systems. To support reproducibility and foster further CV improvements, we made available the full trajectories of the reference unbiased MD simulations that we generated on both systems. We wish that these datasets may serve as benchmark references for existing strategies and future developments. The current validation on two mini-proteins paves the way to study larger and more-complex protein systems with different folding mechanisms or structural characteristics. Assessing the generalizability of our feature set on such systems will be crucial to demonstrate the scalability of the approach.

Looking ahead, this work opens several exciting avenues. The interpretable and portable nature of our CVs makes them highly suitable for integration with modern machine learning techniques to further enhance CV optimization. Applying this methodology to larger proteins will be a key next step to explore phenomena such as domain folding and local conformational changes. The detailed free-energy landscapes generated would provide deeper insights into folding pathways, the nature of transition states, and the thermodynamics and kinetics of misfolding and aggregation processes implicated in disease.

Furthermore, the principles underlying our CV construction strategy, particularly the emphasis on distinguishing native versus non-native interactions and the explicit role of solvent, could be adapted to study more-complex biomolecular processes, such as protein–ligand interactions, RNA folding, and other large-scale conformational transitions. This approach thus offers a valuable and extensible framework for advancing our understanding of the molecular mechanisms governing biological systems.

## Supplementary Material





## Data Availability

The Python script to generate the features, the analysis scripts, and the enhanced sampling simulation input files are available on Github https://github.com/valeriorizzi/FoldingFeatures and on the PLUMES NEST repository.^97^ https://www.plumed-nest.org/eggs/25/019/. The script requires PLUMED,^92,98^ version 2.8 or later. The enhanced sampling simulations are run with GROMACS 2023.^99^ The long unbiased reference trajectory are deposited on Zenodo https://zenodo.org/records/15583283.

## Notes

Additional figures, tables and computational details on the CV building strategy and on enhanced sampling simulations (PDF)
Transparent Peer Review report available (PDF)

## §

For M. Héritier: Institut Pasteur, Université Paris Cité, CNRS UMR 3528, Computational Structural Biology Unit, Paris, France

## References

[1] Calvino, I. Le Città Invisibili [Invisible Cities]; Einaudi: Turin, Italy, 1972.

[2] Dobson C. M., Šali A., Karplus M. (1998). Protein Folding: A
Perspective from
Theory and Experiment. Angew. Chem., Int. Ed..

[3] Dill K. A., Ozkan S. B., Shell M. S., Weikl T. R. (2008). The Protein Folding
Problem. Ann. Rev. Biophys..

[4] Dill K. A., MacCallum J. L. (2012). The Protein-Folding
Problem, 50 Years On. Science.

[5] Rose G. D. (2021). Protein
folding - seeing is deceiving. Protein Sci..

[6] Kocher C. D., Dill K. A. (2024). Origins of life:
The Protein Folding Problem all over
again?. Proc. Natl. Acad. Sci. U. S. A..

[7] Anfinsen C. B., Haber E. (1961). Studies on the Reduction
and Re-formation of Protein Disulfide Bonds. J. Biol. Chem..

[8] Jumper J., Evans R., Pritzel A., Green T., Figurnov M., Ronneberger O., Tunyasuvunakool K., Bates R., Žídek A., Potapenko A. (2021). Highly accurate protein structure prediction
with AlphaFold. Nature.

[9] Chen, S.-j. ; Hassan, M. ; Jernigan, R. L. ; Jia, K. ; Kihara, D. ; Kloczkowski, A. ; Kotelnikov, S. ; Kozakov, D. ; Liang, J. ; Liwo, A. Protein folds vs. protein folding: Differing questions, different challenges. Proc. Natl. Acad. Sci. U.S.A. 2023, 120, e2214423119. 10.1073/pnas.2214423119 36580595 PMC9910419

[10] Frieden C. (2003). The Kinetics
of Side Chain Stabilization during Protein Folding. Biochemistry.

[11] Wang D., Frechette L. B., Best R. B. (2024). On the role of native contact cooperativity
in protein folding. Proc. Natl. Acad. Sci. U.
S. A..

[12] Baldwin R. L., Frieden C., Rose G. D. (2010). Dry molten
globule intermediates
and the mechanism of protein unfolding. Proteins:
Struct., Funct., Bioinf..

[13] Sarkar S. S., Udgaonkar J. B., Krishnamoorthy G. (2013). Unfolding of a Small Protein Proceeds
via Dry and Wet Globules and a Solvated Transition State. Biophys. J..

[14] Wankowicz S. A., Fraser J. S. (2025). Advances in uncovering the mechanisms
of macromolecular
conformational entropy. Nat. Chem. Biol..

[15] Wilson C. B., Yau W.-M., Tycko R. (2024). Experimental
Evidence for Millisecond–Timescale
Structural Evolution Following the Microsecond–Timescale Folding
of a Small Protein. Phys. Rev. Lett..

[16] Lindorff-Larsen K., Piana S., Dror R. O., Shaw D. E. (2011). How Fast-Folding
Proteins Fold. Science.

[17] A
Beccara S., Škrbić T., Covino R., Faccioli P. (2012). Dominant folding pathways of a WW domain. Proc. Natl. Acad. Sci. U.S.A..

[18] A
Beccara S., Fant L., Faccioli P. (2015). Variational scheme
to compute protein reaction pathways using atomistic force fields
with explicit solvent. Phys. Rev. Lett..

[19] Juraszek J., Saladino G., van Erp T. S., Gervasio F. L. (2013). Efficient Numerical
Reconstruction of Protein Folding Kinetics with Partial Path Sampling
and Pathlike Variables. Phys. Rev. Lett..

[20] Levinthal C. (1968). Are there
pathways for protein folding?. J. Chim. Phys..

[21] Levinthal C. (1969). How to fold
graciously. Mössbauer Spectrosc. Biol.
Syst. Proc..

[22] Rose G. D. (2021). Reframing
the Protein Folding Problem: Entropy as Organizer. Biochemistry.

[23] Wolynes P. G., Onuchic J. N., Thirumalai D. (1995). Navigating
the Folding Routes. Science.

[24] Bryngelson J. D., Onuchic J. N., Socci N. D., Wolynes P. G. (1995). Funnels, pathways,
and the energy landscape of protein folding: A synthesis. Proteins: Struct., Funct., Bioinf..

[25] Hardin C., Eastwood M. P., Prentiss M., Luthey-Schulten Z., Wolynes P. G. (2002). Folding funnels: The key to robust
protein structure
prediction. J. Comput. Chem..

[26] Onuchic J. N., Wolynes P. G. (2004). Theory of protein
folding. Curr.
Opin. Struct. Biol..

[27] Shakhnovich E. I., Finkelstein A. V. (1989). Theory of cooperative transitions
in protein molecules.
I. Why denaturation of globular protein is a first-order phase transition. Biopolymers.

[28] Levy Y., Onuchic J. N. (2004). Water and proteins: A love–hate relationship. Proc. Natl. Acad. Sci. U. S. A..

[29] Jungwirth P. (2015). Biological
Water or Rather Water in Biology?. J. Phys.
Chem. Lett..

[30] Clementi C., Nymeyer H., Onuchic J. N. (2000). Topological and energetic factors:
what determines the structural details of the transition state ensemble
and “en-route” intermediates for protein folding? an
investigation for small globular proteins. J.
Mol. Biol..

[31] Valsson O., Tiwary P., Parrinello M. (2016). Enhancing Important Fluctuations:
Rare Events and Metadynamics from a Conceptual Viewpoint. Annu. Rev. Phys. Chem..

[32] Shaw D. E., Deneroff M. M., Dror R. O., Kuskin J. S., Larson R. H., Salmon J. K., Young C., Batson B., Bowers K. J., Chao J. C. (2008). Anton,
a special-purpose machine for molecular dynamics
simulation. Commun. ACM.

[33] Shaw, D. E. ; Adams, P. J. ; Azaria, A. ; Bank, J. A. ; Batson, B. ; Bell, A. ; Bergdorf, M. ; Bhatt, J. ; Butts, J. A. ; Correia, T. Anton 3: Twenty Microseconds of Molecular Dynamics Simulation before Lunch. Presented at SC ’21: The International Conference for High Performance Computing, Networking, Storage and Analysis; ACM, 2021, 10.1145/3458817.3487397.

[34] Shaw D. E., Maragakis P., Lindorff-Larsen K., Piana S., Dror R. O., Eastwood M. P., Bank J. A., Jumper J. M., Salmon J. K., Shan Y. (2010). Atomic-Level Characterization of the Structural Dynamics
of Proteins. Science.

[35] Piana S., Lindorff-Larsen K., Shaw D. E. (2012). Protein folding kinetics and thermodynamics
from atomistic simulation. Proc. Natl. Acad.
Sci. U. S. A..

[36] Piana S., Lindorff-Larsen K., Shaw D. E. (2013). Atomic-level description of ubiquitin
folding. Proc. Natl. Acad. Sci. U. S. A..

[37] Piana S., Robustelli P., Tan D., Chen S., Shaw D. E. (2020). Development
of a Force Field for the Simulation of Single-Chain Proteins and Protein–Protein
Complexes. J. Chem. Theory Comput..

[38] Zhou R. (2003). Trp-cage:
Folding free energy landscape in explicit water. Proc. Natl. Acad. Sci. U. S. A..

[39] Huang X., Hagen M., Kim B., Friesner R. A., Zhou R., Berne B. J. (2007). Replica Exchange
with Solute Tempering: Efficiency
in Large Scale Systems. J. Phys. Chem. B.

[40] Wang L., Friesner R. A., Berne B. J. (2011). Replica
Exchange with Solute Scaling:
A More Efficient Version of Replica Exchange with Solute Tempering
(REST2). J. Phys. Chem. B.

[41] Pan A. C., Weinreich T. M., Piana S., Shaw D. E. (2016). Demonstrating an
Order-of-Magnitude Sampling Enhancement in Molecular Dynamics Simulations
of Complex Protein Systems. J. Chem. Theory
Comput..

[42] Peng X., Zhang Y., Li Y., Liu Q., Chu H., Zhang D., Li G. (2018). Integrating Multiple Accelerated
Molecular Dynamics to Improve Accuracy of Free Energy Calculations. J. Chem. Theory Comput..

[43] Bussi G., Gervasio F. L., Laio A., Parrinello M. (2006). Free-Energy
Landscape for *β* Hairpin Folding from Combined
Parallel Tempering and Metadynamics. J. Am.
Chem. Soc..

[44] Bonati L., Piccini G., Parrinello M. (2021). Deep learning
the slow modes for
rare events sampling. Proc. Natl. Acad. Sci.
U. S. A..

[45] Laio A., Parrinello M. (2002). Escaping free-energy
minima. Proc. Natl. Acad. Sci. U.S.A..

[46] Barducci A., Bussi G., Parrinello M. (2008). Well-Tempered
Metadynamics: A Smoothly
Converging and Tunable Free-Energy Method. Phys.
Rev. Lett..

[47] Invernizzi M., Parrinello M. (2020). Rethinking
Metadynamics: From Bias Potentials to Probability
Distributions. J. Phys. Chem. Lett..

[48] Invernizzi M., Piaggi P. M., Parrinello M. (2020). Unified Approach
to Enhanced Sampling. Phys. Rev. X.

[49] Invernizzi M., Parrinello M. (2022). Exploration
vs Convergence Speed in Adaptive-Bias Enhanced
Sampling. J. Chem. Theory Comput..

[50] Sutto L., D’Abramo M., Gervasio F. L. (2010). Comparing the efficiency of biased
and unbiased molecular dynamics in reconstructing the free energy
landscape of Met-enkephalin. J. Chem. Theory
Comput..

[51] Pietrucci F. (2017). Strategies
for the exploration of free energy landscapes: Unity in diversity
and challenges ahead. Rev. Phys..

[52] Fröhlking T., Aureli S., Gervasio F. L. (2025). Learning
committor-consistent collective
variables. Nat. Comput. Sci..

[53] Rizzi V., Aureli S., Ansari N., Gervasio F. L. (2023). OneOPES, a Combined
Enhanced Sampling Method to Rule Them All. J.
Chem. Theory Comput..

[54] Bussi G. (2014). Hamiltonian
replica exchange in GROMACS: a flexible implementation. Mol. Phys..

[55] Pietrucci F., Laio A. A. (2009). Collective Variable for the Efficient
Exploration of
Protein Beta-Sheet Structures: Application to SH3 and GB1. J. Chem. Theory Comput..

[56] Marinelli F., Pietrucci F., Laio A., Piana S. (2009). A Kinetic Model of
Trp-Cage Folding from Multiple Biased Molecular Dynamics Simulations. PLoS Comput. Biol..

[57] Shaffer P., Valsson O., Parrinello M. (2016). Enhanced,
targeted sampling of high-dimensional
free-energy landscapes using variationally enhanced sampling, with
an application to chignolin. Proc. Natl. Acad.
Sci. U. S. A..

[58] Mendels D., Piccini G., Brotzakis Z. F., Yang Y. I., Parrinello M. (2018). Folding a
small protein using harmonic linear discriminant analysis. J. Chem. Phys..

[59] Spiwok V., Kurečka M., Křenek A. (2022). Collective Variable for Metadynamics
Derived From AlphaFold Output. Front. Mol. Biosci..

[60] Sasmal S., McCullagh M., Hocky G. M. (2023). Reaction Coordinates for Conformational
Transitions Using Linear Discriminant Analysis on Positions. J. Chem. Theory Comput..

[61] Rydzewski J. (2024). Spectral Map
for Slow Collective Variables, Markovian Dynamics, and Transition
State Ensembles. J. Chem. Theory Comput..

[62] Kang P., Trizio E., Parrinello M. (2024). Computing
the committor with the
committor to study the transition state ensemble. Nat. Comput. Sci..

[63] Trizio E., Kang P., Parrinello M. (2025). Everything
everywhere all at once:
a probability-based enhanced sampling approach to rare events. Nat. Comput. Sci..

[64] Chatterjee S., Ray D. (2025). Acceleration with Interpretability:
A Surrogate Model-Based Collective
Variable for Enhanced Sampling. J. Chem. Theory
Comput..

[65] Honda S., Yamasaki K., Sawada Y., Morii H. (2004). 10 Residue Folded Peptide
Designed by Segment Statistics. Structure.

[66] Honda S., Akiba T., Kato Y. S., Sawada Y., Sekijima M., Ishimura M., Ooishi A., Watanabe H., Odahara T., Harata K. (2008). Crystal Structure of
a Ten-Amino Acid Protein. J. Am. Chem. Soc..

[67] Barua B., Lin J. C., Williams V. D., Kummler P., Neidigh J. W., Andersen N. H. (2008). The Trp-cage: optimizing
the stability of a globular
miniprotein. Protein Eng. Des. Select..

[68] Piana S., Laio A. (2007). A bias-exchange approach
to protein folding. J. Phys. Chem. B.

[69] Baldwin R. L., Rose G. D. (2016). How the hydrophobic
factor drives protein folding. Proc. Natl. Acad.
Sci. U. S. A..

[70] Pietrucci F., Marinelli F., Carloni P., Laio A. (2009). Substrate Binding Mechanism
of HIV-1 Protease from Explicit-Solvent Atomistic Simulations. J. Am. Chem. Soc..

[71] Pérez-Conesa S., Piaggi P. M., Parrinello M. (2019). A local fingerprint for hydrophobicity
and hydrophilicity: From methane to peptides. J. Chem. Phys..

[72] Brotzakis Z. F., Limongelli V., Parrinello M. (2019). Accelerating the Calculation of Protein–Ligand
Binding Free Energy and Residence Times Using Dynamically Optimized
Collective Variables. J. Chem. Theory Comput..

[73] Rizzi V., Bonati L., Ansari N., Parrinello M. (2021). The role of
water in host-guest interaction. Nat. Commun..

[74] Ansari N., Rizzi V., Carloni P., Parrinello M. (2021). Water-Triggered,
Irreversible Conformational Change of SARS-CoV-2 Main Protease on
Passing from the Solid State to Aqueous Solution. J. Am. Chem. Soc..

[75] Ansari N., Rizzi V., Parrinello M. (2022). Water regulates the residence time
of Benzamidine in Trypsin. Nat. Commun..

[76] Karrenbrock M., Borsatto A., Rizzi V., Lukauskis D., Aureli S., Luigi Gervasio F. (2024). Absolute Binding
Free Energies with
OneOPES. J. Phys. Chem. Lett..

[77] Febrer
Martinez P., Rizzi V., Aureli S., Gervasio F. L. (2024). Host–Guest
Binding Free Energies à la Carte: An Automated OneOPES Protocol. J. Chem. Theory Comput..

[78] Ding X., Aureli S., Vaithia A., Lavriha P., Schuster D., Khanppnavar B., Li X., Blum T. B., Picotti P., Gervasio F. L., Korkhov V. M. (2024). Structural
basis of
connexin-36 gap junction channel inhibition. Cell Discovery.

[79] Aureli S., Bellina F., Rizzi V., Gervasio F. L. (2024). Investigating Ligand-Mediated
Conformational Dynamics of Pre-miR21: A Machine-Learning-Aided Enhanced
Sampling Study. J. Chem. Inf. Model..

[80] Aureli S., Rizzi V., Piasentin N., Gervasio F. L. (2025). Enhanced Sampling
and Tailored Collective Variables Yield Reproducible Free Energy Landscapes
of Beta-1 Adrenergic Receptor Activation. J.
Chem. Theory Comput..

[81] Welling, M. Fisher Linear Discriminant Analysis; Department of Computer Science, University of Toronto, 2006.

[82] Jha S. K., Udgaonkar J. B. (2009). Direct evidence for a dry molten
globule intermediate
during the unfolding of a small protein. Proc.
Natl. Acad. Sci. U. S. A..

[83] Bonati L., Trizio E., Rizzi A., Parrinello M. (2023). A unified
framework for machine learning collective variables for enhanced sampling
simulations: mlcolvar. J. Chem. Phys..

[84] Fröhlking T., Bonati L., Rizzi V., Gervasio F. L. (2024). Deep learning path-like
collective variable for enhanced sampling molecular dynamics. J. Chem. Phys..

[85] Fröhlking T., Rizzi V., Aureli S., Gervasio F. L. (2024). DeepLNE++ leveraging
knowledge distillation for accelerated multi-state path-like collective
variables. J. Chem. Phys..

[86] Megías A., Contreras Arredondo S., Chen C. G., Tang C., Roux B., Chipot C. (2025). Iterative variational learning of committor-consistent
transition pathways using artificial neural networks. Nature Computational Science.

[87] Mendels D., Piccini G., Parrinello M. (2018). Collective
Variables from Local Fluctuations. J. Phys.
Chem. Lett..

[88] Bonati L., Rizzi V., Parrinello M. (2020). Data-Driven Collective Variables
for Enhanced Sampling. J. Phys. Chem. Lett..

[89] Trizio E., Parrinello M. (2021). From Enhanced
Sampling to Reaction Profiles. J. Phys. Chem.
Lett..

[90] Raucci U., Rizzi V., Parrinello M. (2022). Discover, Sample, and Refine: Exploring
Chemistry with Enhanced Sampling Techniques. J. Phys. Chem. Lett..

[91] Huang D. M., Chandler D. (2000). Temperature and length scale dependence
of hydrophobic
effects and their possible implications for protein folding. Proc. Natl. Acad. Sci. U. S. A..

[92] Tribello G. A., Bonomi M., Branduardi D., Camilloni C., Bussi G. (2014). PLUMED 2: New feathers for an old
bird. Comput.
Phys. Commun..

[93] Byrne A., Williams D. V., Barua B., Hagen S. J., Kier B. L., Andersen N. H. (2014). Folding dynamics and pathways of
the trp-cage miniproteins. Biochemistry.

[94] Chalyavi F., Schmitz A. J., Tucker M. J. (2020). Unperturbed
Detection of the Dynamic
Structure in the Hydrophobic Core of Trp-Cage via Two-Dimensional
Infrared Spectroscopy. J. Phys. Chem. Lett..

[95] Hradiská H., Kurečka M., Beránek J., Tedeschi G., Višňovský V., Křenek A., Spiwok V. (2024). Acceleration of Molecular Simulations
by Parametric Time-Lagged tSNE Metadynamics. J. Phys. Chem. B.

[96] Juraszek J., Bolhuis P. G. (2008). Rate constant and reaction coordinate
of Trp-cage folding
in explicit water. Biophys. J..

[97] Bonomi M., Bussi G., Camilloni C., Tribello G. A. (2019). Promoting transparency
and reproducibility in enhanced molecular simulations. Nat. Methods.

[98] Tribello G. A., Bonomi M., Bussi G., Camilloni C., Armstrong B. I., Arsiccio A., Aureli S., Ballabio F., Bernetti M., Bonati L. (2025). PLUMED Tutorials: A
collaborative, community-driven learning ecosystem. J. Chem. Phys..

[99] Abraham M.
J., Murtola T., Schulz R., Páll S., Smith J. C., Hess B., Lindahl E. (2015). GROMACS: High performance
molecular simulations through multi-level parallelism from laptops
to supercomputers. SoftwareX.

