# Supplementary material for: The Arch from the Stones: Understanding Protein Folding Energy Landscapes via Bioinspired Collective Variables
Source: J Phys Chem Lett. 2025 Sep 8;16(37):9636–45. doi: 10.1021/acs.jpclett.5c02079 (PMC12451740; doi:10.1021/acs.jpclett.5c02079)
Supplement: Supplementary file 1 [file jz5c02079_si_001.pdf]

# Supporting Information

## The Arch from the Stones: Understanding Protein Folding Energy Landscapes via Bio-inspired Collective Variables

Valerio Rizzi[1,2,3,∇,**], Margaux Héritier[1,2,3,5,∇], Nicola Piasentin[1,2,3], Simone Aureli[1,2,3], and Francesco Luigi Gervasio[1,2,3,4,*]

[1]University of Geneva, Rue Michel Servet 1, 1211, Genève 4, Switzerland
[2]School of Pharmaceutical Sciences and Institute of Pharmaceutical Sciences of Western Switzerland (ISPSO)
[3]Swiss Institute of Bioinformatics, University of Geneva, 1206, Genève, Switzerland
[5]Department of Chemistry, University College London, London, WC1E 6BT, United Kingdom
[4]Current address: Institut Pasteur, Université Paris Cité, CNRS UMR 3528, Computational Structural Biology Unit, Paris, France.
[*]francesco.gervasio@unige.ch
[**]valerio.rizzi@unige.ch

[∇]V.R. and M.H. contributed equally to the manuscript

# Materials and methods

## H-bond feature definition

An H-bond is an electrostatic interaction between a hydrogen atom $H$ that is covalently bound to a more electronegative *Donor* atom $D$ and an H-bond *Acceptor* atom $A$ that must be electronegative and must possess a lone pair of electrons, covalently bound to atom $Z$ (see Fig. 1 (a) in the main text). In this section, we focus on a single H-bond between atoms $H_i$ and $A_j$ extracted from an unbiased trajectory of one of the end states (indexes are omitted for simplicity in Fig. 1 (a) in the main text and in the text below) and go through the corresponding H-bond feature $F_{HA}^{\mathrm{HB}}$ construction. The selection and filtering process of the H-bonds is discussed below.

Hydrogen bonding arises from the overlap between $H$'s orbital and the sp$^3$ hybrid orbitals of $A$, which exhibit spatial anisotropy. While the strength of an H-bond is mainly modulated by the $\overline{HA}$ distance, it also depends on the orientation of its constituent atoms. We monitor the former through a coordination function $C_{HA}$ whose value ranges from 1 when the bond is present to 0 at longer distances. We use a long-range switching function (SWF) of the following functional form

$$C_{HA} = \frac{1-\left(\frac{r}{r_0}\right)^n}{1-\left(\frac{r}{r_0}\right)^m} \tag{S1}$$

where $r = \overline{HA}$, $r_0 = 3\,\text{Å}$, $n = 6$ and $m = 8$ (see Fig. S1, in blue).

The atomic orientation is encoded by two functions $\Gamma_{\mathrm{H/A}}$ (see Fig. 1 (a) in the main text) which are equal to 1 when the angles $\widehat{DHA}/\widehat{ZAH}$, centred on atoms $H$ and $A$ respectively, are flat and decrease as the angles bend [1, 2]. These functions are simply defined as a ratio of distances

$$\Gamma_{\mathrm{H}} = \frac{\overline{DA}}{\overline{HD}+\overline{HA}} \tag{S2}$$

and

$$\Gamma_{\mathrm{A}} = \frac{\overline{HZ}}{\overline{HA}+\overline{AZ}}. \tag{S3}$$

We define variable

$$C'_{HA} = C_{HA} \cdot \Gamma_{\mathrm{H}} \cdot \Gamma_{\mathrm{A}} \tag{S4}$$

as an indicator of H-bond strength that takes into account both bond length and angular contributions (see Fig. S2 for illustrative plots of $C'_{HA}$ as a function of bond length and angle).

In an ideal $\alpha$-helix or $\alpha$-sheet backbone, native hydrogen bonds align perfectly, maximizing their strength. However, thermal fluctuations, solvent exposure and environmental interactions introduce deviations, weakening the bonds and increasing their flexibility. $C'_{HA}$ is a powerful tool for classifying H-bonds according to their length and orientation. If its average value in the examined unbiased trajectory is above an arbitrary threshold of 0.6, we consider the H-bond to be strong and name it *hard*. Otherwise, we name a wobblier H-bond *soft*. For hard H-bonds, we bring forward to the H-bond feature definition the full angular information contained in $F_{HA}^{\mathrm{HB,H}} = C'_{HA}$, while for soft H-bonds we revert to a simpler form $F_{HA}^{\mathrm{HB,S}} = C_{HA}$.

We further classify an HB feature in terms of its specialisation in capturing a bond in either the folded or the unfolded state. To do so, we calculate the mean value of the H-bond feature $F_{HA}^{\mathrm{HB,H/S}}$ during the folded and unfolded unbiased trajectory. If the feature has a higher mean value in the folded state, it captures a native H-bond and we call it $F_{HA}^{\mathrm{HB,H/S,F}}$, otherwise we call it $F_{HA}^{\mathrm{HB,H/S,U}}$. Naturally, as the folded state is more structured, the number of $F_{HA}^{\mathrm{HB,H/S,F}}$ features tends to be larger than the number of $F_{HA}^{\mathrm{HB,H/S,U}}$.

For an H-bond feature to work efficiently in driving folding and unfolding events, it must contain information not only about a native bond being present, but also about other competing non-native bonds

being absent. As a native H-bond contact of a protein cleaves during protein unfolding, energetic considerations dictate that it must be instantly replaced by a non-native interaction with other protein H-bond donors/acceptors or with water molecules. This bond replacement mechanism can be intuitively included into a feature by using opposite signs [3] for the two categories of native and non-native bonds.

We evaluate non-native interactions with protein atoms only for the folded-state-focused features $F_{HA}^{\mathrm{HB,H/S,F}}$ with the aim to boost the capability of the resulting H-bond CV to strongly distinguish the folded state from the variety of semi-folded metastable states that resemble it. Such a need is not present in the unfolded basin that naturally includes a number of very different structures. Sharply determining the formation of a non-native contact (NNC) and filtering it from the surrounding noisy environment is crucial. The formation of NNCs can take place on both ends of the HB, namely either around atom $H$ or $A$. To take both into account, we first dynamically determine two virtual atom positions along the $\overline{DH}$ and the $\overline{ZA}$ vectors, 2.5 Å away from $D$ and $Z$ (see Fig. 1 (a) in the main text). We call these virtual atoms $V_H$ and $V_A$, respectively.

To evaluate the coordination number $C_i$ between $V_H$ and all possible non native protein acceptor atoms and between $V_A$ and all possible non native protein donor atoms, we use the following short-ranged SWF

$$\mathrm{C}_i = \alpha_i \sum_{j}^{r_{ij}<r_{\mathrm{NL}}} \frac{\left( \frac{1-\left(\frac{r_{ij}}{r_0}\right)^n}{1-\left(\frac{r_{ij}}{r_0}\right)^m} - \frac{1-\left(\frac{d_{\mathrm{MAX}}}{r_0}\right)^n}{1-\left(\frac{d_{\mathrm{MAX}}}{r_0}\right)^m} \right)}{\left(1 - \frac{1-\left(\frac{d_{\mathrm{MAX}}}{r_0}\right)^n}{1-\left(\frac{d_{\mathrm{MAX}}}{r_0}\right)^m}\right)} \tag{S5}$$

where $\alpha_i$ is a normalization factor and $r_{ij}$ represents the distance between a virtual atom $i$, either $V_H$ or $V_A$, and all non-native protein acceptor/donor atoms $j$. This SWF goes smoothly to zero at distance $r_{ij} = d_{\mathrm{MAX}}$ and is null in the buffer zone $d_{\mathrm{MAX}} < r_{ij} < r_{\mathrm{NL}}$ where $r_{\mathrm{NL}}$ is the neighbour list radius. We set $r_0 = 3.5\,\text{Å}$, $n = 2$, $m = 10$, $d_{\mathrm{MAX}} = 5\,\text{Å}$, $r_{\mathrm{NL}} = 8\,\text{Å}$ (see Fig. S1, in red), and update the neighbour list every 20 steps. The short-rangedness of the SWF is essential in picking up the signal of specific NNCs arising in close vicinity to $i$ and in reducing the noise from atoms further apart.

In the case of non-native interactions with water, we calculate them for both folded-state-focused and unfolded-state-focused features, but only for hard H-bonds $F^{\mathrm{HB,H,F}}$ that present a well defined position where a water molecule would stay. We evaluate the coordination number between $V_H$ and all water oxygen atoms and between $V_A$ and all water hydrogen atoms using the same SWF from Eq. S5. As before, we set $r_0 = 3.5\,\text{Å}$, $n = 2$, $m = 10$, $d_{\mathrm{MAX}} = 5\,\text{Å}$, $r_{\mathrm{NL}} = 8\,\text{Å}$ (see Fig. S1, in red), and update the neighbour list every 20 steps.

Ideally, we would like the value of an HB feature to go from about 1 when the native contact is present to about $-1$ when a NNC replaces it. To achieve so, the NNC coordination number must be normalised so that its value compensate and does not exceed the one of the native contact. In the case of a NNC calculated between $V_H$ and water oxygen atoms ($C_{\mathrm{WAT,V_H}}$) we use a normalisation factor $\alpha_i = 1/8$, while in the case between $V_H$ and protein acceptor atoms ($C_{\mathrm{NNC,V_H}}$) we use a normalisation factor of 1. On the other hand, for NNC calculated between $V_A$ and the more numerous water hydrogen atoms ($C_{\mathrm{WAT,V_A}}$) we use a normalisation factor of 1/16 and finally in the case between $V_A$ and protein donor atoms ($C_{\mathrm{NNC,V_A}}$) we use a normalisation factor of 1/2.

All in all, the final form of a folded-state-specialised H-bond feature is

$$F_{HA}^{\mathrm{HB,H,F}} = C_{HA} \cdot \Gamma_{\mathrm{H}} \cdot \Gamma_{\mathrm{A}} - C_{\mathrm{NNC,V_H}} - C_{\mathrm{NNC,V_A}} - C_{\mathrm{WAT,V_A}} - C_{\mathrm{WAT,V_H}} \tag{S6}$$

for a hard H-bond and

$$F_{HA}^{\mathrm{HB,S,F}} = C_{HA} - C_{\mathrm{NNC,V_H}} - C_{\mathrm{NNC,V_A}} \tag{S7}$$

for a soft H-bond.

Instead, an unfolded-state-specialised feature is

$$F_{HA}^{\mathrm{HB,H,U}} = C_{HA} \cdot \Gamma_{\mathrm{H}} \cdot \Gamma_{\mathrm{A}} - C_{\mathrm{WAT,V_A}} - C_{\mathrm{WAT,V_H}} \tag{S8}$$

for a hard H-bond or simply

$$F_{HA}^{\mathrm{HB,S,U}} = C_{HA} \tag{S9}$$

for a soft H-bond.

In summary, the difference between folded-state-specialised features $F_{HA}^{\mathrm{HB,H/S,F}}$ and unfolded-state-specialised features $F_{HA}^{\mathrm{HB,H/S,U}}$ lies in the presence of the NNC terms with respect to protein acceptors/donors only in the folded-state-specialised case. These terms help to sharpen the resolution of the CV in the key region in close vicinity to the folded state, while the same would not be helpful in the more unstructured unfolded basin.

## Side chain packing feature definition

The second feature that we craft, $F^{\mathrm{SC}}$, specialises in capturing side chain packing through the relative orientation of the residues. It is complementary to the H-bond feature as it is aimed at distinguishing states that may share H-bonds contacts and secondary structure, but have a different degree of compactness. Its components describe individual native contacts between side chains and distinguish by construction spurious non-native contacts that characterise metastable states.

We describe the side chains at the mesoscale level with the distances of their centre of mass (see Fig. 1 (b) in the main text). In the special case of side chain-free glycine residues, the side chain COM is replaced by its C$\alpha$ atom. In analogy with our treatment of $F^{\mathrm{HB}}$, we focus on the case of one contact between hypothetical residues $X$ and $Y$ and leave the contact selection and filtering process to the next section.

First, we transform the $\overline{XY}$ distance into a contact $C_{XY}$ through a SWF of the form contained in Eq. S1. We use $r_0 = 8$ Å, $n = 4$ and $m = 8$. This SWFs is rather long-ranged as it is focused on picking up a single specific contact between $X$ and $Y$, even when they are far apart (see Fig. 1 (b) in the main text and the curve in green in Fig. S1). Its long-rangedness is fundamental to pull $X$ and $Y$ together when going from the unfolded basin to the folded basin in enhanced sampling. Depending on the mean value of $C_{XY}$ in the unbiased trajectories, the resulting feature would be either folded-state specialised $F_{XY}^{\mathrm{SC,F}}$ or unfolded-state specialised $F_{XY}^{\mathrm{SC,U}}$.

To better distinguish metastable states from the end states and reduce overall degeneracy in the frustrated space of side chain packing [4], we also include information about NNC, with a negative sign in a similar fashion as we do for $F^{\mathrm{HB}}$. During the trajectories, we define a virtual atom $V_{XY}$ at the geometrical centre between $X$ and $Y$. We calculate the contact between $V_{XY}$ and all other side chain COMs beside $X$ and $Y$ (see Fig. 1 (b) in the main text). To reduce noise, we use here the short-ranged SWF from Eq. S5 with $r_0 = 3.5$ Å, $n = 2$, $m = 10$, $d_{\mathrm{MAX}} = 5$ Å, $r_{\mathrm{NL}} = 8$ Å, and a neighbour list update every 20 steps. We call the resulting contact $C_{\mathrm{NNC},V_{XY}}$.

The final form of the side chain packing feature is then

$$F^{\mathrm{SC,F}} = C_{XY} - C_{\mathrm{NNC},V_{XY}} \tag{S10}$$

for a folded-state-specialised feature and

$$F^{\mathrm{SC,U}} = C_{XY} \tag{S11}$$

for an unfolded-state-specialised feature.

## Switching function additional information

Here, we illustrate the behaviour of the switching function embedded into the CVs. In Fig. S1, we show in blue the switching function from Eq. S1 for $r_0 = 3$ Å, $n = 6$ and $m = 8$, in green the same function for $r_0 = 8$ Å, $n = 4$ and $m = 8$, and in red the switching function from Eq. S5 for $r_0 = 3.5$ Å, $n = 2$, $m = 10$, $d_{\mathrm{MAX}} = 5$ Å, and $r_{\mathrm{NL}} = 8$ Å. In Fig. S2, we focus on the behaviour of the $C'_{HA}$ descriptor for different acceptor-hydrogen distances given a donor-hydrogen-acceptor angle and vice versa, to illustrate the discriminative power of $C'_{HA}$. The reader can refer to Eq. S2-S4 and Fig. 1 (a) of the main text for further details.

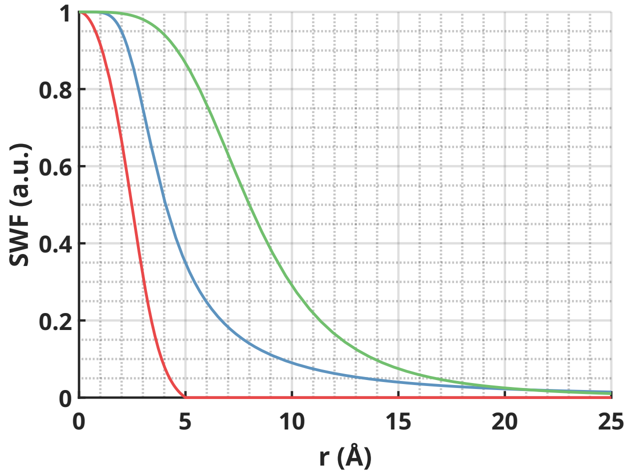


Figure S1: In blue and green, switching function from Eq. S1, in red switching function from Eq. S5. The corresponding parameters are reported both in the text.

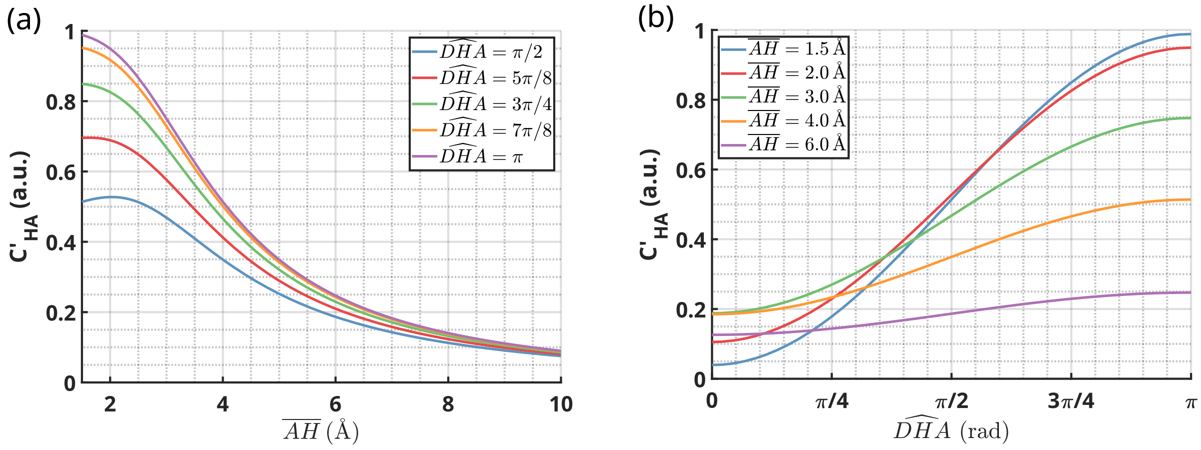


Figure S2: $C'_{HA}$ as defined in Eq. S4 as a function of (a) the hydrogen-acceptor distance for different donor-hydrogen-acceptor angles and (b) vice versa.

## Filtering features

Up to now, we have described in detail how to design features assuming that we already know which ones are relevant. Here we take a step back and show how to automatically select them. This selection process is crucial for reducing the very large space of possible features and picking out only the most relevant ones, thus filtering unwanted noise in the CVs to be generated.

The starting point is two short unbiased trajectories of the folded state F and the unfolded state U, indicatively 100 ns each totalling at least 1000 independent configurations.

Our leading criterion is to rank and filter the features according to their discriminative power between the two end states via an LDA-like score. Through successive steps, we apply a filtering strategy to progressively identify the most relevant H-bond and side-chain packing features and discard those that do not contain a sufficient level of information.

For the H-bond features, we first perform a rough preliminary selection by gathering all the possible

combinations $ij$ between any hydrogen atom $H_i$ bound to an H-bond donor $D_i$ and any H-bond acceptor $A_j$ and calculating their distance. At first we keep only the combinations where a long-lived H-bond exists by retaining only the features in which the mean distance in either the folded/unfolded basin $\mu^{F/U}_{H_iA_j} < 4$ Å. Out of these, we keep only those where $|\mu^{F}_{H_iA_j} - \mu^{U}_{H_iA_j}| > 0.1$ Å, hence discarding all the H-bonds that are equally stable in both end states. These filtering steps significantly reduce the number of H-bonds feature candidates by discarding all contacts that do not generate an H-bond in either state or that generate one in both states.

To classify whether an H-bond is hard or soft, we evaluate its corresponding descriptors $C_{H_iA_j}$ and $C'_{H_iA_j}$ as defined in Eq. S1 and Eq. S4, respectively. If the average value of $C'_{H_iA_j}$ in either of the end states is above a threshold value of 0.6, then the H-bond is classified as hard and is retained. If not, we revert to the simpler contact $C_{H_iA_j}$. If its average value $C_{H_iA_j} > 0.6$ on either state, the H-bond is classified as soft and is also retained. If not, the H-bond is discarded.

Out of the remaining features, we evaluate their standard deviation over the end states $\sigma^{F/U}_{H_iA_j}$ and calculate their discriminative power

$$S_{H_iA_j} = (\mu^{F}_{H_iA_j} - \mu^{U}_{H_iA_j})^2 / (\sigma^{F}_{H_iA_j} + \sigma^{U}_{H_iA_j}) \tag{S12}$$

according to an LDA-like criterion. We keep features with a discriminative power $S_{H_iA_j} > 0.3$.

Features are further classified according to the sign of $\mu^{F}_{H_iA_j} - \mu^{U}_{H_iA_j}$, with a positive sign indicating features that are folded-specialised and, conversely, a negative sign for unfolded-specialised.

In the calculation of NNC that lead to protein-protein hydrogen bonds ($C_{\mathrm{NNC,V_H}}$ and $C_{\mathrm{NNC,V_A}}$), we include in the summation all possible donor-acceptor combinations, excluding the non native contacts that are in close vicinity to the given native one in the folded state i.e. with an average distance less than 4 Å.

For the side chain packing features, the filtering procedure is analogous and simpler. We take into account only non adjacent side chain contacts, excluding those between residues $X_i$ and $Y_{i\pm1}$. Out of these features, we keep all those that have a value $\mu^{F/U}_{X_iY_j} = C_{X_iY_j} > 0.6$ in either state and a discriminative power $S_{X_iY_j} > 0.3$.

## Additional hydration CVs

In the OneOPES strategy, additional CVs can be biased in higher order replicas in order to accelerate supplementary degrees of freedom and boost convergence. In this context, given the importance of the protein solvation and the hydrophobic core collapse, we decide to extract relevant water CVs according to the LDA-like criterion in Eq. S12.

We first calculate the coordination number $C_{\mathrm{WAT,PROT}_i}$ between all protein heavy atoms and the water oxygen atoms with the SWF from Eq. S5. We use $r_0 = 3$ Å, $n = 6$, $m = 10$, $d_{\mathrm{MAX}} = 10$ Å, $r_{\mathrm{NL}} = 15$ Å and update the neighbour list every 20 steps. Following a filtering strategy analogous to the one presented above, we determine their discriminative power $S_i$ from Eq. S12 and rank them. We take the 7 highest ranked CVs and bias them progressively along the OneOPES replica ladder.

## Computational details

We use the open-source molecular dynamics software GROMACS 2023 [5] patched with PLUMED2 version 2.9.1 [6, 7] to perform all simulations. The Python script to generate the features, the analysis scripts and the enhanced sampling simulation input files are available on the paper repository on Github https://github.com/valeriorizzi/FoldingFeatures.

### System preparation for Chignolin

The Chignolin miniprotein has been extensively used for benchmarking new methods aiming at studying fast-folding proteins. In analogy, here we use the double mutant CLN025, shown in Fig. 1 (c) in the

main text, which has two tyrosine mutations (G1Y and G10Y) [8, 9]. The protein is obtained mutating the corresponding wild-type (PDB ID: 1UAO [8]).

Our Python script identifies five hard and eleven soft H-bonds significant in the folded state, and two soft H-bonds significant in the unfolded state. The packing is driven by eight side-chain contacts that are all significant in the folded state.

We use the CHARMM22* force field. The simulation box contains 1907 TIP3P water molecules and two sodium ions to achieve charge neutrality. We use periodic boundary conditions and the particle-mesh-Ewald (PME) method to treat Coulomb long-range electrostatic interactions, while a cut-off distance of 1.0 nm is applied for Coulomb and van der Waals short-range interactions. After 100 ns of simulations of both folded and unfolded states, we run the OneOPES MultiCV simulation in the NVT ensemble with a time step of 2 fs, using the V-rescale thermostat at 340K [10]. The unbiased run is 300 $\mu$s long, split into three 100 $\mu$s long independent trajectories, while the five OPES and OneOPES runs are each 1 $\mu$s long.

The free energy results that we present are an average over the five independent trajectories. Free energies are calculated by applying standard reweighting on replica 0 using the script `FES_from_Reweighting.py` from Refs. [11, 12]. Free energy differences between the folded and the unfolded basins are estimated on the RMSD over the C$\alpha$ atoms. We skip the first 20 ns of the trajectories and use $\sigma = 0.01$ nm, 120 bins and a definition of the basins of $0.015 < \text{RMSD} < 0.2$ nm and $0.2 < \text{RMSD} < 0.85$ nm.

In the OPES simulations, we bias $s^{\text{HB}}$ and $s^{\text{SC}}$ with `OPES_METAD_EXPLORE` with a `BARRIER` of 20 kJ/mol a deposition `PACE` of 50000 steps (100 ps). `SIGMA` values are the standard deviation of each CV that is extracted from the unbiased folded trajectory.

To test abridged CVs where we would switch off non-native contacts, we modified individual features by excluding different negative terms. In the case of excluding only non-native contacts with the protein, Eqs. S6, S7 and S10 become

$$F_{HA}^{\text{HB,H,F}} = C_{HA} \cdot \Gamma_{\text{H}} \cdot \Gamma_{\text{A}} - C_{\text{WAT,V}_\text{A}} - C_{\text{WAT,V}_\text{H}} \tag{S13}$$

$$F_{HA}^{\text{HB,S,F}} = C_{HA} \tag{S14}$$

$$F^{\text{SC,F}} = C_{XY}. \tag{S15}$$

Instead, in the case of switching off only non-native contacts with water molecules, Eqs. S6 and S8 become

$$F_{HA}^{\text{HB,H,F}} = C_{HA} \cdot \Gamma_{\text{H}} \cdot \Gamma_{\text{A}} - C_{\text{NNC,V}_\text{H}} - C_{\text{NNC,V}_\text{A}} \tag{S16}$$

$$F_{HA}^{\text{HB,H,U}} = C_{HA} \cdot \Gamma_{\text{H}} \cdot \Gamma_{\text{A}}. \tag{S17}$$

Finally, switching off all non-native contacts, Eqs. S6, S8, S7 and S10 become

$$F_{HA}^{\text{HB,H,F}} = C_{HA} \cdot \Gamma_{\text{H}} \cdot \Gamma_{\text{A}} \tag{S18}$$

$$F_{HA}^{\text{HB,H,U}} = C_{HA} \cdot \Gamma_{\text{H}} \cdot \Gamma_{\text{A}} \tag{S19}$$

$$F_{HA}^{\text{HB,S,F}} = C_{HA} \tag{S20}$$

$$F^{\text{SC,F}} = C_{XY}. \tag{S21}$$

For the OneOPES simulations, the main bias that appears on all replicas is analogous to the one of the OPES simulations (`BARRIER` of 20 kJ/mol, `PACE` of 50000 steps, same `SIGMA`). In higher replicas, starting from replica 1, we sample the multithermal ensemble thanks to OPES MultiThermal, with a `PACE` of 1000. The temperature range for each replica starting from 1 to 7 is: [339K, 342K], [338K, 345K], [337K, 350K], [336K, 360K], [335K, 380K], [330K, 400K], [320K, 420K]. In these replicas, we also bias the water coordination around selected protein heavy atoms. In total we have 4 carbon atoms and 3 oxygen or nitrogen atoms. Replica 1 presents an additional bias on the water coordination of the atom with highest discriminative power, replica 2 independently bias 2 of them until replica 7 that has a bias on all of them. This additional `OPES_METAD_EXPLORE` bias is deposited with a `PACE` of 100000 steps (200 ps) and a `BARRIER` of 3 kJ/mol. Similarly to the main CVs, `SIGMA` corresponds to the standard deviation in the folded state.

**System preparation for TRP-cage**

For the TRP-cage system, we focus on the most studied mutant, namely the Tc10b K8A mutant [13]. The protein is described by the DES-Amber SF1.0 force field [14] and is solvated in a cubic box with TIP4PD water that contains three sodium ions and two chlorine ions to match Ref. [14]. After equilibration, the unbiased run and the enhanced sampling simulations are run in the NPT ensemble with a time step of 2 fs. The temperature is set at 320K and controlled by the V-rescale thermostat [10], while pressure is set at 1 bar and controlled by the C-rescale barostat [15] with a compressibility of $4.5 \times 10^{-5}$ bar$^{-1}$. The unbiased run is 200 $\mu$s long, while the five OneOPES runs are each 1 $\mu$s long.

Our Python script identifies nine hard and twelve soft H-bonds significant in the folded state, and six soft H-bonds significant in the unfolded state. The packing is driven by twenty-six side-chain contacts that are all significant in the folded state.

The free energy results that we present are again an average over the five independent trajectories. Free energy differences between the folded and the unfolded basins are estimated on the RMSD over the C$\alpha$ atoms. We skip the first 20 ns of the trajectories and use $\sigma = 0.02$ nm, 150 bins and a definition of the basins of $0.03 <$ RMSD $< 0.5$ nm and $0.5 <$ RMSD $< 2.0$ nm.

In the OneOPES simulations, we bias $s^{\mathrm{HB}}$ and $s^{\mathrm{SC}}$ as main CVs with `OPES_METAD_EXPLORE` on all replicas. The `SIGMA` values are the standard deviation of each CV as extracted from the unbiased folded trajectory. The bias is deposited with a `PACE` of 50000 steps (100 ps) and a `BARRIER` of 30 kJ/mol.

Starting from replica 1, we include OPES MultiThermal, with a `PACE` of 1000. The temperature range for each replica starting from 1 to 7 is: [319K, 325K], [317K, 332K], [315K, 345K], [310K, 360K], [305K, 380K], [298K, 400K], [290K, 420K]. We also bias as additional CVs the water coordination of four carbon and three oxygen/nitrogen atoms. This additional `OPES_METAD_EXPLORE` bias is deposited with a `PACE` of 100000 steps and a `BARRIER` of 3. Similarly to the main CVs, `SIGMA` corresponds to the standard deviation in the folded state.

## Computational analysis

Cluster analysis on the TRP-Cage trajectories is performed using Visual Molecular Dynamics (VMD)'s *measure cluster* routine [16]. An RMSD threshold value of 1.5 Å is selected considering the number of generated cluster families and the similarity of protein conformations within a cluster family. For each basin, we adapt the PLOT NA routine of the "Drug Discovery Tool" (DDT) to estimate the frequency of occurrence of contacts [17] and assess TRP-Cage's intra-protein interactions. We set a neighbouring cut-off value of 3.0 Å between interacting residues.

# Results

## Chignolin

In this section, we report additional details of Chignolin's folding process, extracted from the 300 $\mu$s-long unbiased trajectory, the 5 OPES simulations, and the 5 OneOPES simulations. First, we provide further insights into the unbiased simulations, such as the sampling of the RMSD auxiliary CV (see Fig. S3). Then, we performed a thorough assessment of the total energies across the 300 $\mu$s-long unbiased trajectory to ensure the thermodynamic consistency of the simulation (see Fig. S4). Moving on to the biased simulations, we first show the free energy of simulations where we use abridged CVs in which we switched off non-native contacts with the protein or with water (see S5). Then, we display the exploration of the $s^{\mathrm{HB}}$, $s^{\mathrm{SC}}$, and RMSD CVs in both the OPES and OneOPES trajectories (see Fig. S6 and Fig. S7, respectively) supporting our claim that the conformational ensemble is thoroughly and consistently explored in both biased and unbiased runs. We conclude this section with a direct comparison of the 2D FES along the $s^{\mathrm{HB}}$ and $s^{\mathrm{SC}}$ CVs obtained from the OPES and OneOPES enhanced sampling simulations (see Fig. S8).

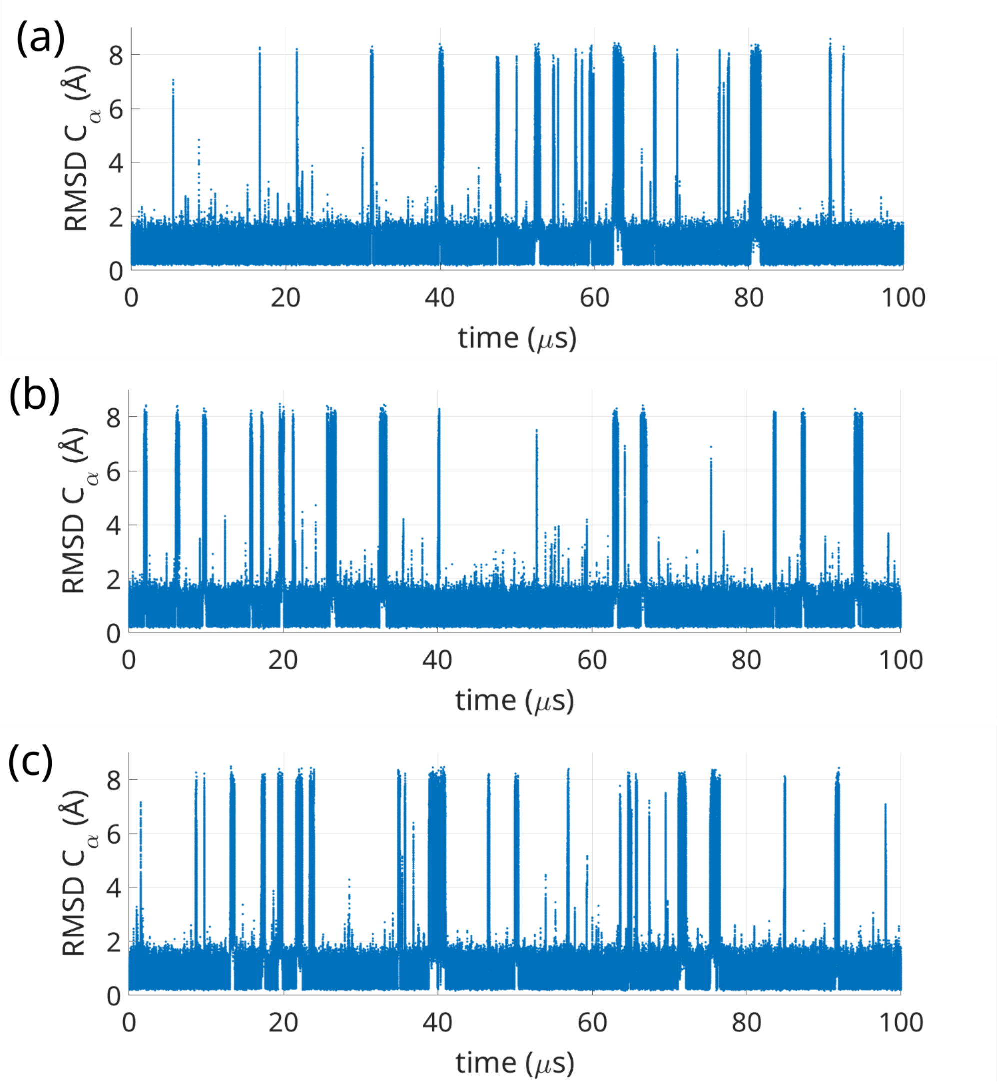


Figure S3: **Folding dynamics of TRP-Cage in unbiased simulations. a-c)** Exploration of the RMSD CV across 300 $\mu$s of unbiased MD simulations. The whole trajectory is made of three independent replicas of 100 $\mu$s each, whose dynamics is displayed in panels (a), (b), and (c).

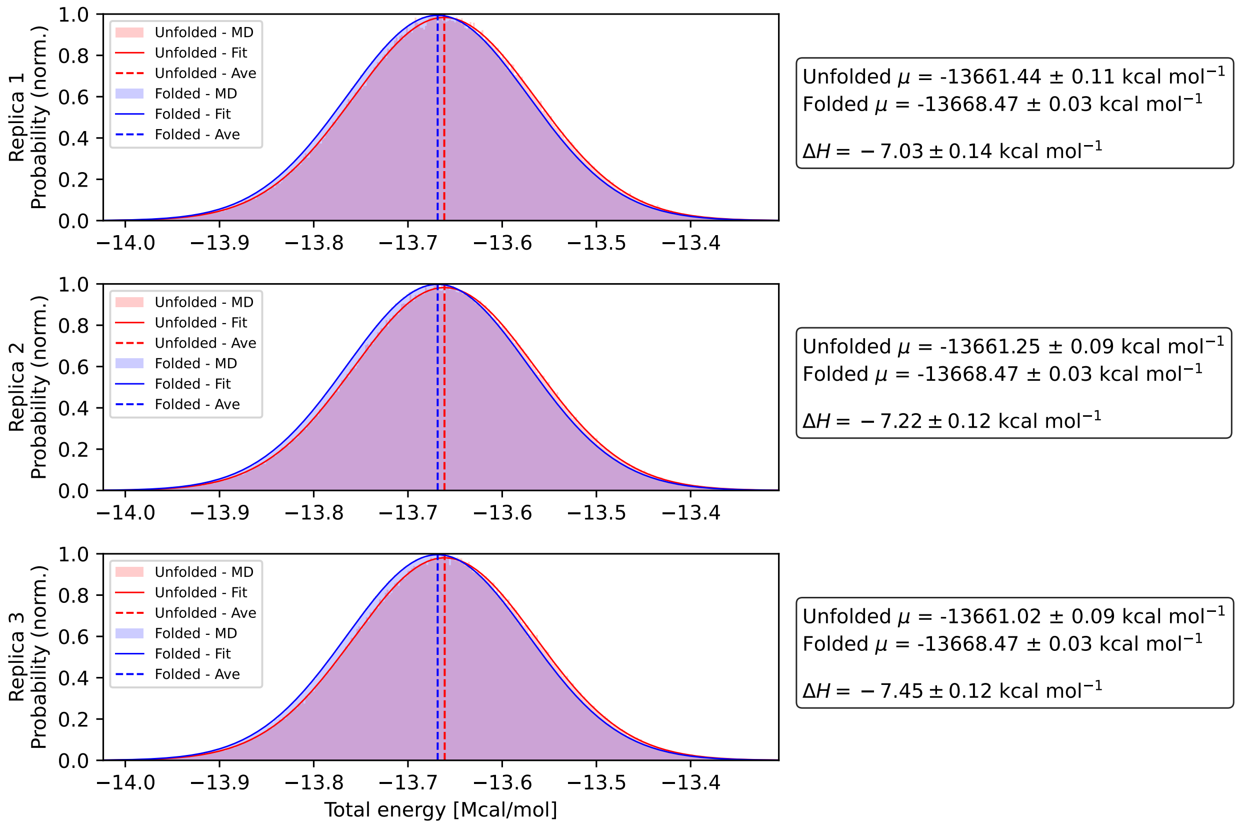


Figure S4: **Distribution of total energies (enthalpies) sampled during the 300 $\mu$s-long unbiased MD simulation of Chignolin.** Histogram representations of enthalpy collected throughout the trajectory, split into the three independent replicas of 100 $\mu$s each. Each distribution is overlaid with a Gaussian fit to highlight the absence of long-timescale drifts or multimodal behaviour.

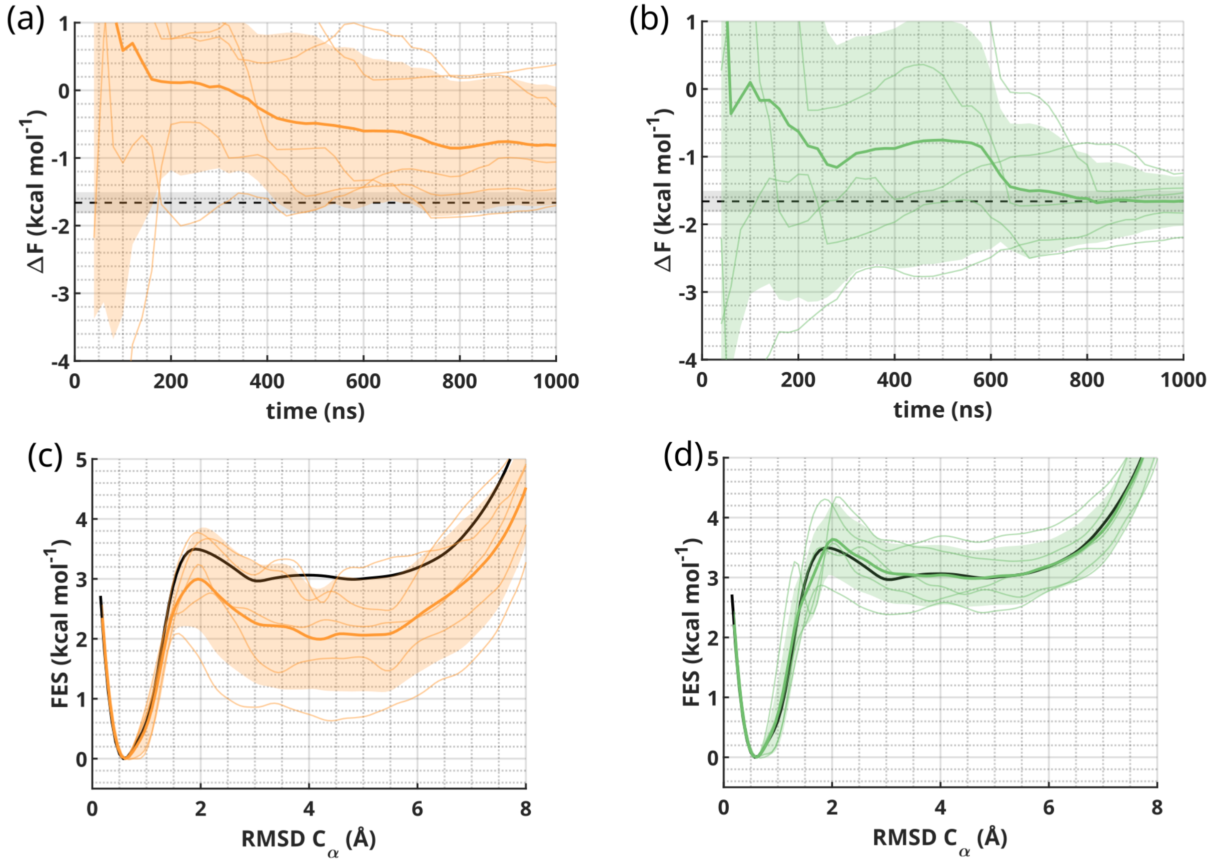


Figure S5: **Chignolin folding abridged CVs results. (a-b)** Chignolin free-energy difference $\Delta F$ between the folded and unfolded states over time from five independent OPES trajectories where non-native contacts with the protein **(a)** or with water **(b)** were switched off, respectively. The reference value is displayed as a black dashed line, the trajectories' average value in solid colour, orange and green, respectively, and the standard deviation in semi-transparency. **(c-d)** 1D FES as a function of the RMSD over the C$\alpha$ atoms for OPES simulations where non-native contacts with the protein **(c)** or with water **(d)** were switched off, respectively. In solid black line we show the reference FES, in solid colour, orange and green, respectively, we report the average of the five replicas, and in semi-transparency their standard deviation.

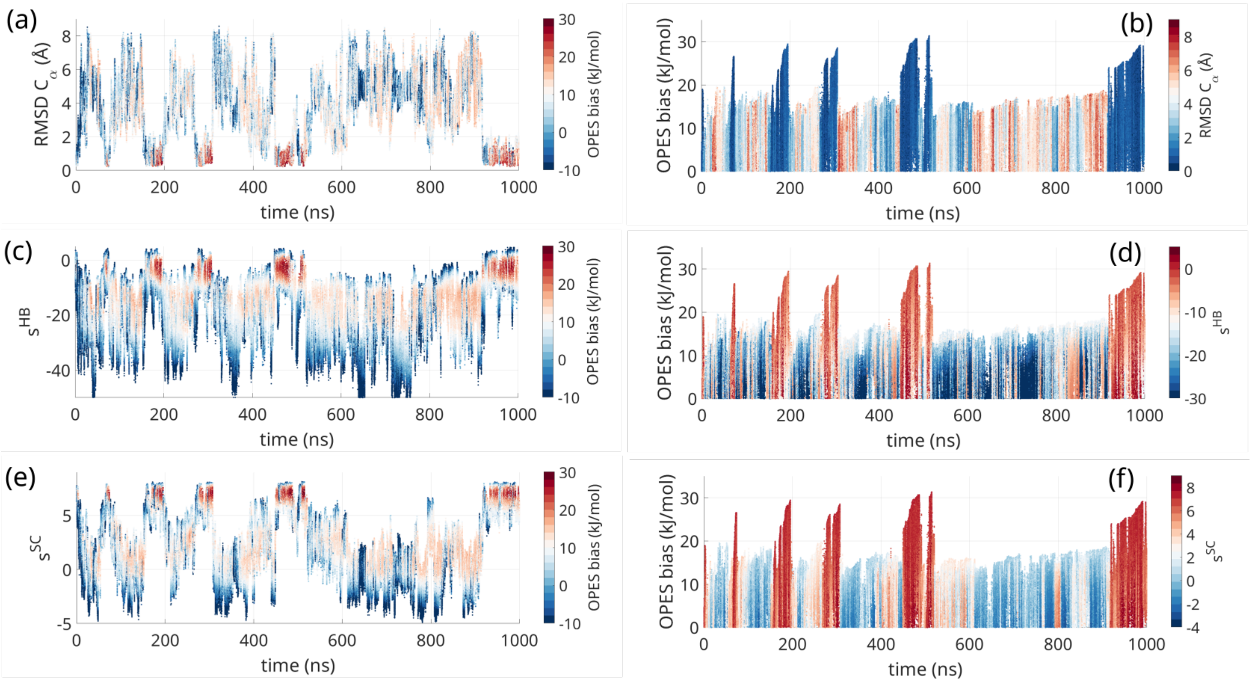


Figure S6: **RMSD and bias evolution during a Chignolin OPES simulation.** **a)** Time evolution of the RMSD CV, coloured by the OPES bias potential. **b)** Time evolution of the OPES bias potential, coloured by RMSD. **c)** Time evolution of the $s^{HB}$ CV, coloured by the OPES bias potential. **d)** Time evolution of the OPES bias potential, coloured by the $s^{HB}$ CV. **e)** Time evolution of the $s^{SC}$ CV, coloured by the OPES bias potential. **f)** Time evolution of the OPES bias potential, coloured by the $s^{SC}$ CV.

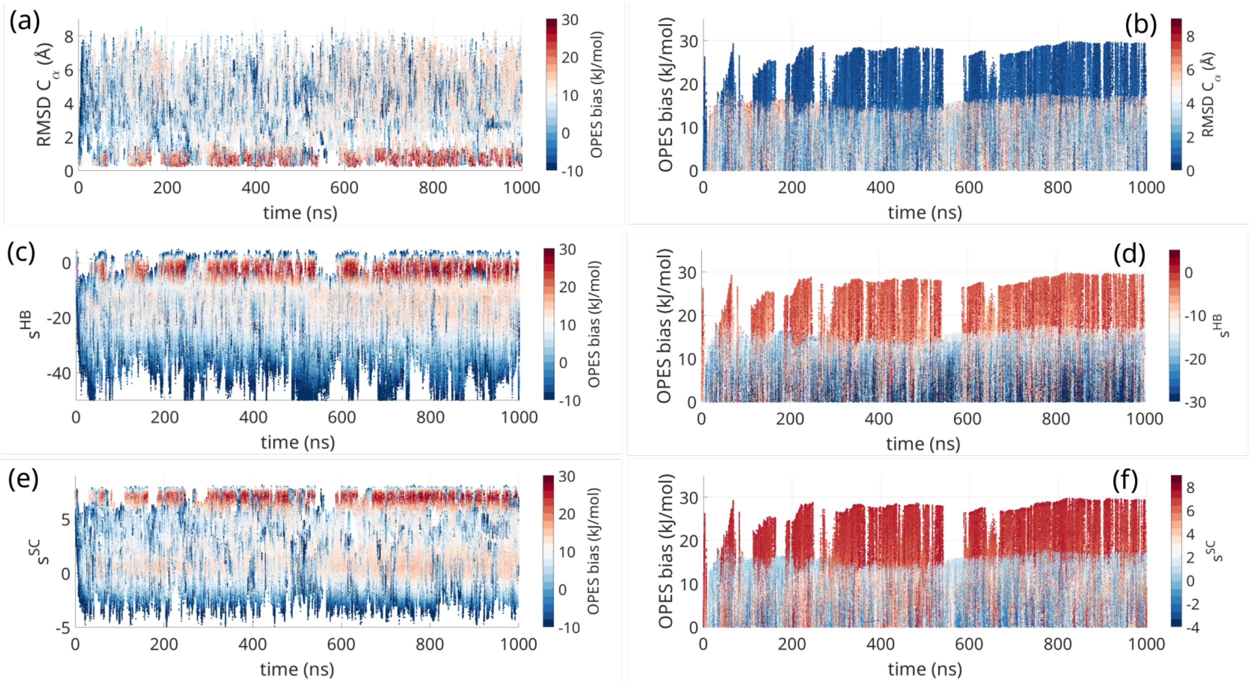


Figure S7: **RMSD and bias evolution during a Chignolin OneOPES simulation. a)** Time evolution of the RMSD CV, coloured by the OPES bias potential. **b)** Time evolution of the OPES bias potential, coloured by RMSD. **c)** Time evolution of the $s^{HB}$ CV, coloured by the OPES bias potential. **d)** Time evolution of the OPES bias potential, coloured by the $s^{HB}$ CV. **e)** Time evolution of the $s^{SC}$ CV, coloured by the OPES bias potential. **f)** Time evolution of the OPES bias potential, coloured by the $s^{SC}$ CV.

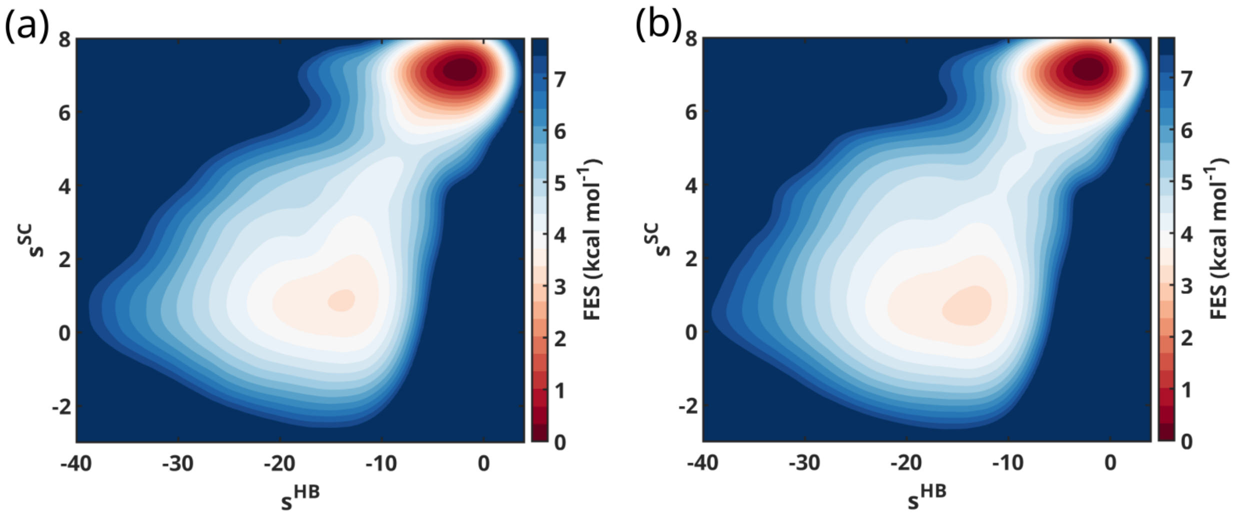


Figure S8: **Chignolin 2D FES as a function of the $s^{HB}$ and $s^{SC}$ CVs. a)** Free-energy surface obtained from the OPES simulations. **b)** Free-energy surface obtained from the OneOPES simulations.

## TRP-Cage

In this section, we report additional details of TRP-Cage folding process, extracted from both the 200 $\mu$s-long unbiased trajectory and the 5 OneOPES simulations. In details, we plan to show additional insights about the sampling of both primary (i.e., $s^{HB}$ and $s^{SC}$) and auxiliary (e.g. RMSD and AlphaRMSD) CVs (see Fig. S9, and Fig. S10), corroborating our assumption that the whole conformational manifold is well sampled in both biased and unbiased runs (see Fig. S11). Lastly, we present further information on the TRP-Cage basins extracted from the 2D FES of the unbiased reference (see Fig. S12) and of OneOPES (see Fig. S13).

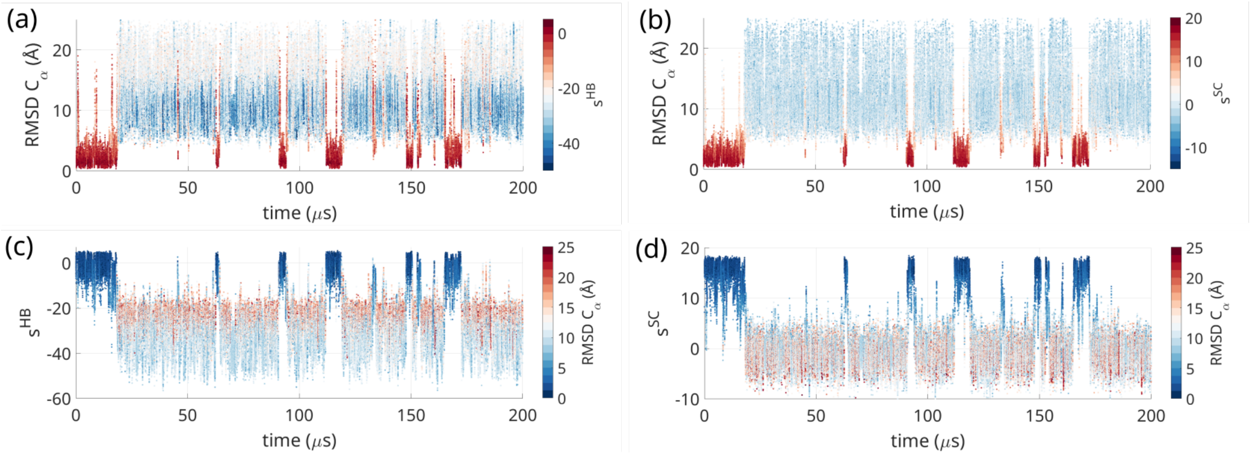


Figure S9: **Folding dynamics of TRP-Cage in an unbiased simulation. a-b)** Time evolution of the RMSD, coloured by the $s^{HB}$ (a) and $s^{SC}$ (b) CVs, respectively. **c)** Time evolution of the $s^{HB}$ CV, coloured by RMSD. **d)** Time evolution of the $s^{SC}$ CV, coloured by RMSD.

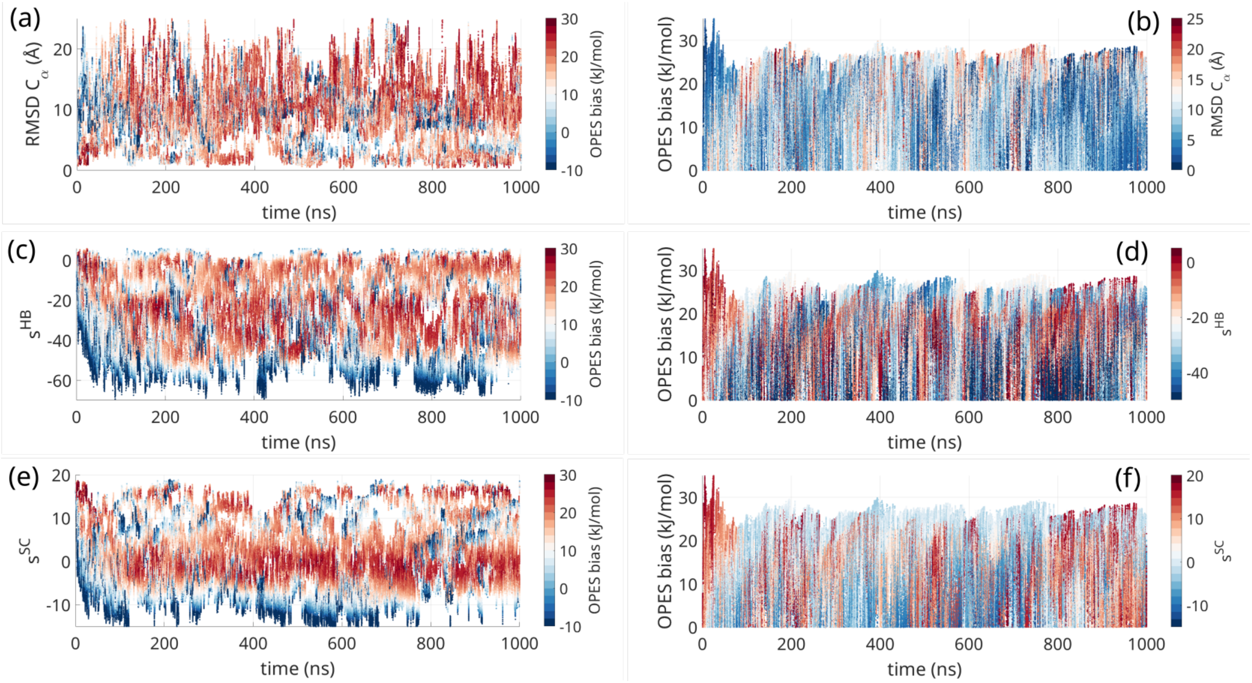


Figure S10: **RMSD and bias evolution of replica 0 during a TRP-cage OneOPES simulation. a)** Time evolution of the RMSD CV, coloured by the OPES bias potential. **b)** Time evolution of the OPES bias potential, coloured by RMSD. **c)** Time evolution of the $s^{HB}$ CV, coloured by the OPES bias potential. **d)** Time evolution of the OPES bias potential, coloured by the $s^{HB}$ CV. **e)** Time evolution of the $s^{SC}$ CV, coloured by the OPES bias potential. **f)** Time evolution of the OPES bias potential, coloured by the $s^{SC}$ CV.

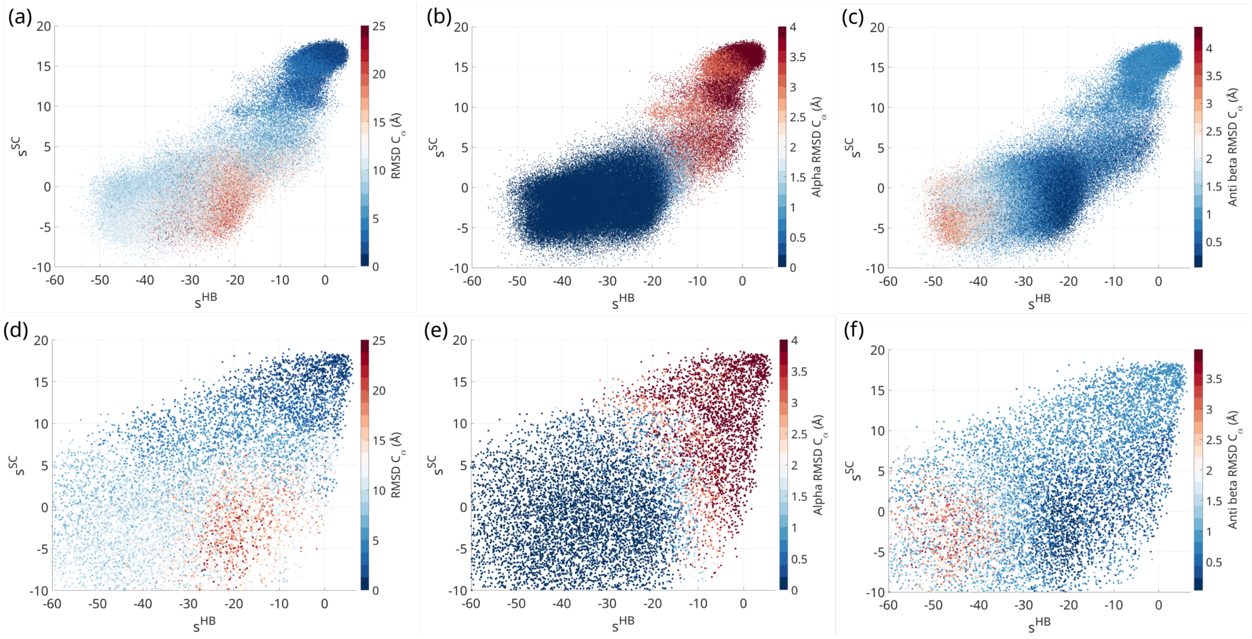


Figure S11: **Comparison of the CV space sampling during unbiased and OneOPES simulations of TRP-Cage. a–c)** Distribution of sampled configurations in the $s^{HB}$–$s^{SC}$ collective variable space during the 200 $\mu$s unbiased MD simulation. **d–f**) Distribution of sampled configurations in the $s^{HB}$–$s^{SC}$ CV space during a OneOPES simulation. For the sake of clarity, points are coloured by different structural descriptors to highlight conformational diversity: panels (a) and (d) are coloured by their RMSD values; panels (b) and (e) are coloured according to their helical content (i.e., AlphaRMSD CV); panels (c) and (f) are coloured according to their $\beta$-structure content (i.e., AntiBeta CV).

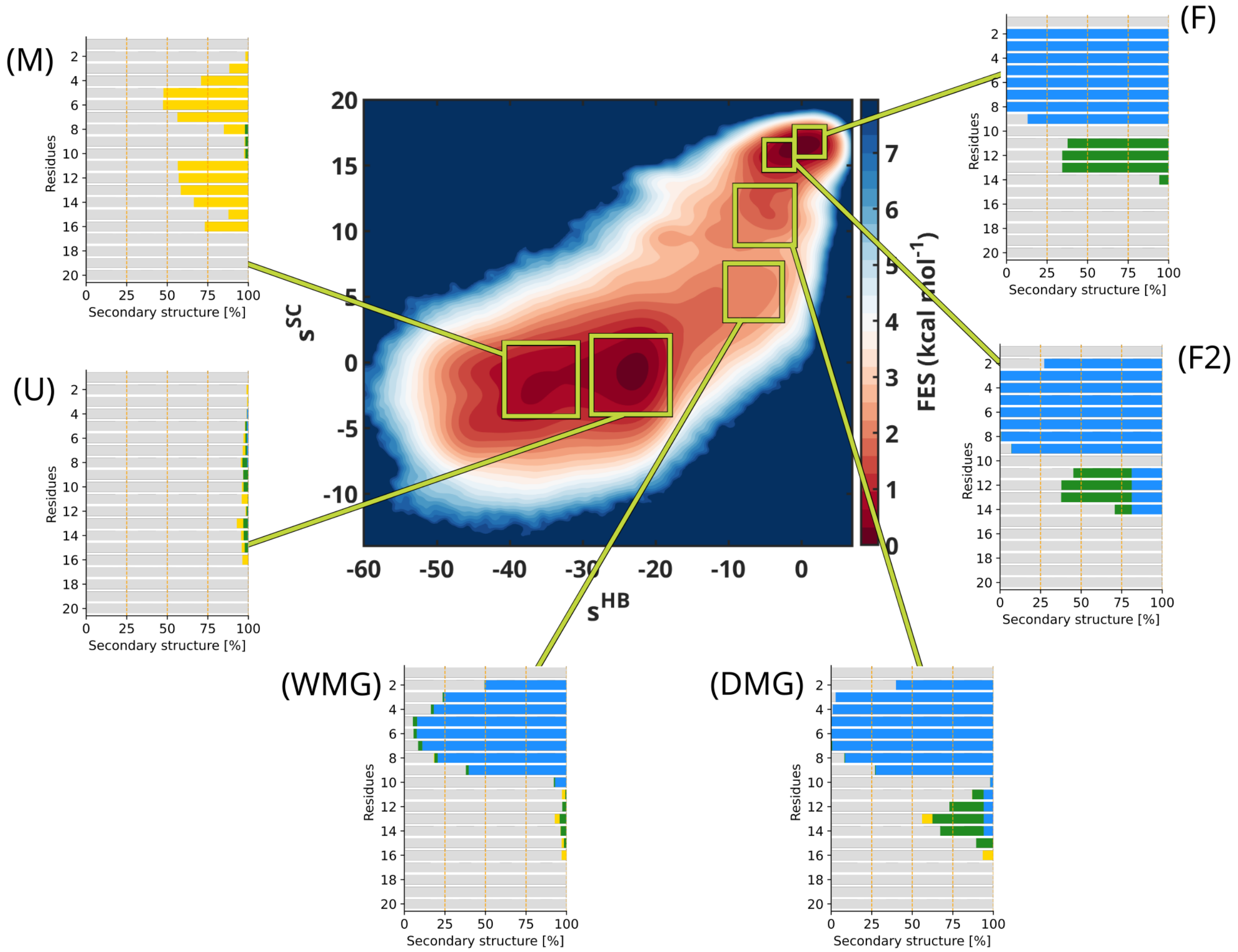


Figure S12: **Trp-Cage folding landscape analysis of the unbiased reference trajectory.** In analogy with Fig. 4 in the main text, we repeat the analysis on the secondary structure of the six conformational ensembles (M, U, WMG, DMG, F2 and F) on our reference unbiased trajectory. For each ensemble, we present a histogram summarising the secondary structure frequency for each amino acid. $\alpha$-helices are coloured in blue, $3_{10}$-helices are coloured in green, $\pi$-helices in magenta, $\beta$-helices in yellow, and random coils in grey.

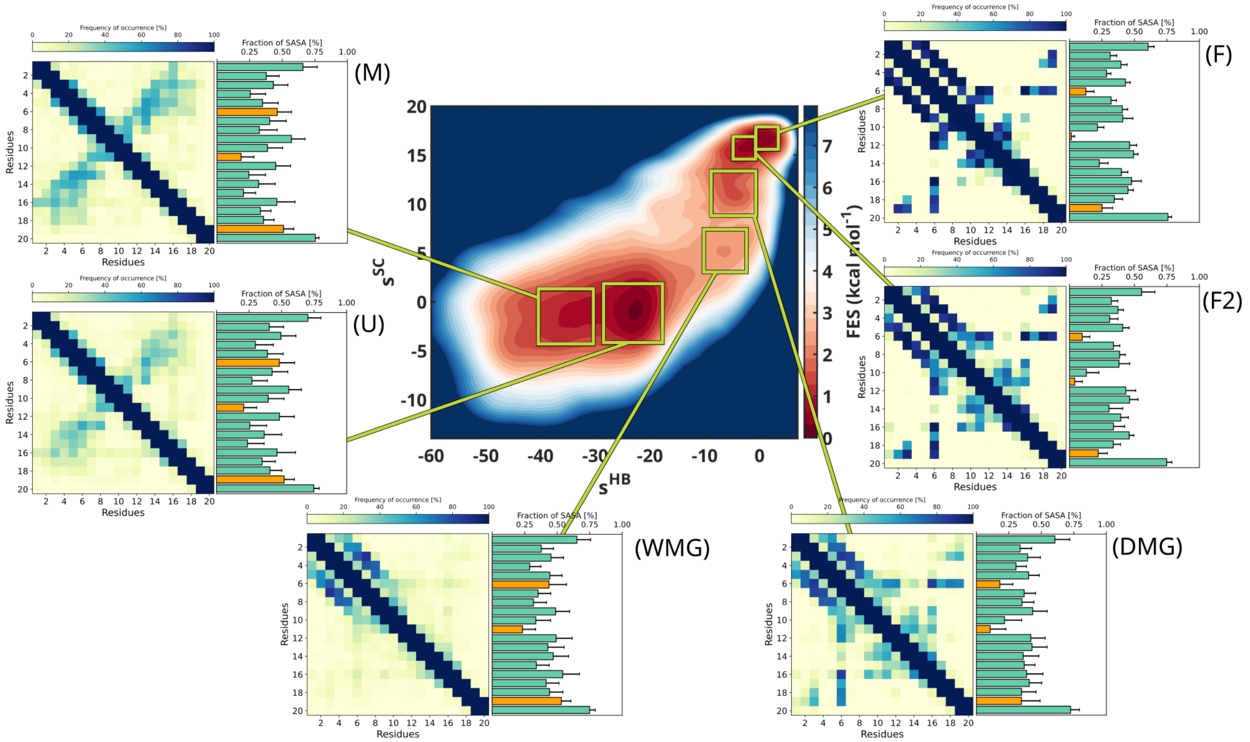


Figure S13: **Additional analysis carried out on the minima extracted from Trp-Cage 2D FES.** For each basin, we display both a frequency map of the inter-residue contacts and the residue-wise histogram of the Solvent-Exposed Surface Area (SASA). In particular, SASA values are normalized with respect to those of the fully solvated amino acids. The contact maps are coloured according to the colour bars over them. In the SASA histograms, bars are shown in green, except for residues W6, G11, and P19, which are highlighted in orange to emphasize their role in hydrophobic core formation

# References


[1] P. Raiteri, A. Laio and M. Parrinello, *Physical Review Letters*, 2004, **93**, 087801.

[2] D. Branduardi, F. L. Gervasio, A. Cavalli, M. Recanatini and M. Parrinello, *Journal of the American Chemical Society*, 2005, **127**, 9147–9155.

[3] D. Polino and M. Parrinello, *The Journal of Physical Chemistry B*, 2019, **123**, 6851–6856.

[4] E. I. Shakhnovich and A. V. Finkelstein, *Biopolymers*, 1989, **28**, 1667–1680.

[5] M. J. Abraham, T. Murtola, R. Schulz, S. Páll, J. C. Smith, B. Hess and E. Lindahl, *SoftwareX*, 2015, **1-2**, 19–25.

[6] G. A. Tribello, M. Bonomi, D. Branduardi, C. Camilloni and G. Bussi, *Computer Physics Communications*, 2014, **185**, 604–613.

[7] G. A. Tribello, M. Bonomi, G. Bussi, C. Camilloni, B. I. Armstrong, A. Arsiccio, S. Aureli, F. Ballabio, M. Bernetti, L. Bonati, S. G. H. Brookes, Z. F. Brotzakis, R. Capelli, M. Ceriotti, K.-t. Chan, P. Cossio, S. Dasetty, D. Donadio, B. Ensing, A. L. Ferguson, G. Fraux, J. D. Gale, F. L. Gervasio, T. Giorgino, N. S. M. Herringer, G. M. Hocky, S. E. Hoff, M. Invernizzi, O. Languin-Cattoën, V. Leone, V. Limongelli, O. Lopez-Acevedo, F. Marinelli, P. Febrer Martinez, M. Masetti, S. Mehdi, A. Michaelides, M. H.

Murtada, M. Parrinello, P. M. Piaggi, A. Pietropaolo, F. Pietrucci, S. Pipolo, C. Pritchard, P. Raiteri, S. Raniolo, D. Rapetti, V. Rizzi, J. Rydzewski, M. Salvalaglio, C. Schran, A. Seal, A. Shayesteh Zadeh, T. F. D. Silva, V. Spiwok, G. Stirnemann, D. Sucerquia, P. Tiwary, O. Valsson, M. Vendruscolo, G. A. Voth, A. D. White and J. Wu, *The Journal of Chemical Physics*, 2025, **162**, 092501.

[8] S. Honda, K. Yamasaki, Y. Sawada and H. Morii, *Structure*, 2004, **12**, 1507–1518.

[9] S. Honda, T. Akiba, Y. S. Kato, Y. Sawada, M. Sekijima, M. Ishimura, A. Ooishi, H. Watanabe, T. Odahara and K. Harata, *Journal of the American Chemical Society*, 2008, **130**, 15327–15331.

[10] G. Bussi, D. Donadio and M. Parrinello, *The Journal of Chemical Physics*, 2007, **126**, 014101.

[11] M. Invernizzi and M. Parrinello, *The Journal of Physical Chemistry Letters*, 2020, **11**, 2731–2736.

[12] M. Invernizzi and M. Parrinello, *Journal of Chemical Theory and Computation*, 2022, **18**, 3988–3996.

[13] B. Barua, J. C. Lin, V. D. Williams, P. Kummler, J. W. Neidigh and N. H. Andersen, *Protein Engineering Design and Selection*, 2008, **21**, 171–185.

[14] S. Piana, P. Robustelli, D. Tan, S. Chen and D. E. Shaw, *Journal of Chemical Theory and Computation*, 2020, **16**, 2494–2507.

[15] M. Bernetti and G. Bussi, *The Journal of Chemical Physics*, 2020, **153**, 114107.

[16] W. Humphrey, A. Dalke and K. Schulten, *Journal of Molecular Graphics*, 1996, **14**, 33–38.

[17] S. Aureli, D. Di Marino, S. Raniolo and V. Limongelli, *Bioinformatics*, 2019, **35**, 5328–5330.
