# Supplementary material for: The Arch from the Stones: Understanding Protein Folding Energy Landscapes via Bioinspired Collective Variables
Source: J Phys Chem Lett. 2025 Sep 8;16(37):9636–45. doi: 10.1021/acs.jpclett.5c02079 (PMC12451740; doi:10.1021/acs.jpclett.5c02079)
Supplement: Supplementary file 2 [file jz5c02079_si_002.pdf]

jz-2025-02079u.R1

Name: Peer Review Information for "The Arch from the Stones: Understanding Protein Folding Energy Landscapes via Bio-inspired Collective Variables"

First Round of Reviewer Comments

Reviewer: 1

Comments to the Author

The manuscript presents a new approach for design of collective variables (CVs) for enhanced sampling simulations of miniprotein folding. The manuscript is original and well written. There are few issues to be addressed:

- CV degeneracy is discussed in the introduction, but the definition is missing. I understand degeneracy of CVs as the fact that certain value(s) of CV(s) correspond to two or more different states, relevant to the studied process. Inability of CVs to resolve metastable states cited by authors is, in my opinion, the result, not the cause, of degeneracy. Correct me if I'm wrong.

- P6L30 comparing potential energy of hydrogen bond with free energy of folding can be confusing for researchers who are not experienced in molecular physics. I agree with the point of authors but I believe it can be rephrased to avoid confusion of potential and free energy.

- P7L15 it would be nice to explicitly state how long are short simulations, so that the reader does not have to go to SI.

- Authors should indicate how uncertainties of predictions were calculated.

- It would be nice to report numbers of folding/unfolding events in biased and unbiased simulations.

Reviewer: 2

Comments to the Author

In this manuscript, the authors present a new methodology to develop collective variables for efficiently capturing protein folding/unfolding in molecular dynamics simulations. Overall, this is an interesting work with considerable novelty. The manuscript is well written and clear. I believe that this work will be of interest to the readership of JPCL, especially people from the molecular simulation field. I only have a few minor comments listed below that I would ask the authors to address. Once they have satisfactorily addressed them, I can strongly recommend that the manuscript is accepted for publication in JPCL.

- If possible, it would be good if the author can slightly more details about the new CVs that they have developed in this work. At the moment, it is hard to fully them from the main text, and one needs to look at the SI. I can understand that due to space constrain the authors cannot have a full discussion about the CVs in the main part. Still, it would be good if they can add a bit more details in the main text.

- The authors define and use a few non-standard abbreviations, such as NNC (non native contacts), DOF, WMG, and DMG, those usage I find to hinder the readability of the manuscript, as the reader might be forced to look back to find the definitions. In particular, the NNC and DOF abbreviations are only used once in the main text from what I can see, thus, I don't see a need to define in the main part (it can still be used in the SI). Thus, in general, I would recommend that the author reconsider the usage of non-standard abbreviations where it might hinder the readability of the manuscript, especially the NNC and DOF abbreviations.

- For both the Chignolin and Trp-Cage system, the author perform long (~100 microsecond) unbiased simulations to get reference data for the free energy difference. To gauge the accuracy of this reference data, I would ask the author to give further information about these reference simulations in the main manuscript. For example, how many unfolding/folding events do they observe in the simulations, what is the average unfolding and folding times in the simulations (as this would need to be considerably shorter than the total simulation time to get accurate estimates of the free energy differences). Also, it would be good to report the temperature used in the simulations, as they are run at slightly elevated temperature to have a larger number unfolding/folding events (similar as was done in the Ref 17). I would ask that the author add this further information to the main manuscript.

- Page 8, Figure 2: For the OneOPES simulation in Fig 2(c), is the simulation time shown on the x-axis, simulation time per replica or cumulative simulation time? This is an important distinction to have far comparison between the two simulation methods. Thus, I would ask the author to clarify this, and note this in the caption and text.

- Page 9, Figure 3, for the 2D FESs in panels (c) and (f), the s^{HB} CV ranges from -60 to around 0, while the s^{SC} CV ranges from -15 to 20. For the s^{HB} CV, why is only showing negative values and why is the folded state around value of 0? Given the definition of the CV, I would think that the folded state would have higher positive values than exhibited in the 2D FESs.

- Page 14, Ref 4 and 5 are the same citation.

Author's Response to Peer Review Comments:

Dear Journal of Physical Chemistry Letters Editor,
Dear Editor,

We would like to thank the reviewers for their constructive comments and questions. We have revised and improved the manuscript in line with their recommendations and performed new simulations. Below, for each question or comment, we report the reviewers' comment in black and our response in blue. We have also highlighted in blue the changes that we have made in the revised version of the manuscript.

Kind regards,

Francesco Luigi Gervasio on behalf of all authors

Reviewer: 1

The manuscript presents a new approach for design of collective variables (CVs) for enhanced sampling simulations of miniprotein folding. The manuscript is original and well written. There are few issues to be addressed:

- CV degeneracy is discussed in the introduction, but the definition is missing. I understand degeneracy of CVs as the fact that certain value(s) of CV(s) correspond to two or more different states, relevant to the studied process. Inability of CVs to resolve metastable states cited by authors is, in my opinion, the result, not the cause, of degeneracy. Correct me if I'm wrong.

We agree with the reviewer that degeneracy in a CV is indeed the cause of bad resolution. We have clarified this point in the revised manuscript at page 3 by adding an explicit definition of degeneracy and better explained the concept.

- P6L30 comparing potential energy of hydrogen bond with free energy of folding can be confusing for researchers who are not experienced in molecular physics. I agree with the point of authors but I believe it can be rephrased to avoid confusion of potential and free energy.

We have clarified this aspect by explicitly highlighting the *potential* energy contribution of a single hydrogen bond in the revised main text.

- P7L15 it would be nice to explicitly state how long are short simulations, so that the reader does not have to go to SI.

We have added an indication of the typical length of the short unbiased simulations - about 100ns - needed for the workflow.

- Authors should indicate how uncertainties of predictions were calculated.

We have added a short statement on page 7 of the revised main text and a longer discussion to the SI where we explain in detail the calculation of errors. All of our biased simulations are run in 5 independent copies. Our free energy estimates and their errors are the mean and the standard deviation of such simulations. We have added to the paper's Github repository the scripts that we used to calculate such quantities.

- It would be nice to report numbers of folding/unfolding events in biased and unbiased simulations.

For the unbiased simulations, we now report in the revised main text the total number of back and forth folding/unfolding events, 76 for Chignolin and 26 for TRPcage. We follow the dual-cutoff approach from Ref. [1] with a time window of 10 ns. In both cases, we use the RMSD over the Cα atoms as a standard discriminant. For Chignolin, we set a folding threshold of 1.5 Å and an unfolding threshold of 2.5 Å. For TRPcage, the thresholds are 4.0 Å and 6.0 Å, respectively. We also added the corresponding Matlab script to the paper's Github repository.

For the biased simulations, especially in combination with replica-exchange, the number of folding/unfolding events is an ill defined quantity that cannot be properly quantified with the same reliable dual-cutoff approach. In that case, transitions can occur either because of local bias deposition on a replica or because of coordinate exchange that here we attempt every 20ps. Very fast back and forth transitions can deceivingly occur on the picosecond timescale, but without a longer relaxation phase it is impossible to filter noise from complete folding/unfolding events. Therefore, to avoid misleading the readers regarding this delicate aspect, we decided to avoid discussing the number of transitions in biased simulations in the revised manuscript.

Reviewer: 2

In this manuscript, the authors present a new methodology to develop collective variables for efficiently capturing protein folding/unfolding in molecular dynamics simulations. Overall, this is an interesting work with considerable novelty. The manuscript is well written and clear. I believe that this work will be of interest to the readership of JPCL, especially people from the molecular simulation field. I only have a few minor comments listed below that I would ask the authors to address. Once they have satisfactorily addressed them, I can strongly recommend that the manuscript is accepted for publication in JPCL.

- If possible, it would be good if the author can slightly more details about the new CVs that they have developed in this work. At the moment, it is hard to fully them from the main text, and one needs to look at the SI. I can understand that due to space constrain the authors cannot have a full discussion about the CVs in the main part. Still, it would be good if they can add a bit more details in the main text.

We agree with the reviewer on this point, but unfortunately the space constraints of a letter are quite strict and our manuscript is already on the length limit. Even if we would like more of the technical discussion of the CV construction to appear in the main text, space

constraints force us to keep it in the SI, leaving a shorter discussion to the main text.

- The authors define and use a few non-standard abbreviations, such as NNC (non native contacts), DOF, WMG, and DMG, those usage I find to hinder the readability of the manuscript, as the reader might be forced to look back to find the definitions. In particular, the NNC and DOF abbreviations are only used once in the main text from what I can see, thus, I don't see a need to define in the main part (it can still be used in the SI). Thus, in general, I would recommend that the author reconsider the usage of non-standard abbreviations where it might hinder the readability of the manuscript, especially the NNC and DOF abbreviations.

In the revised manuscript, we removed the NNC and the DOF abbreviations. We kept the definition of NNC in the SI as it is present in the formulas that describe the CVs constructions.

- For both the Chignolin and Trp-Cage system, the author perform long ($\sim$100 microsecond) unbiased simulations to get reference data for the free energy difference. To gauge the accuracy of this reference data, I would ask the author to give further information about these reference simulations in the main manuscript. For example, how many unfolding/folding events do they observe in the simulations, what is the average unfolding and folding times in the simulations (as this would need to be considerably shorter than the total simulation time to get accurate estimates of the free energy differences). Also, it would be good to report the temperature used in the simulations, as they are run at slightly elevated temperature to have a larger number unfolding/folding events (similar as was done in the Ref 17). I would ask that the author add this further information to the main manuscript.

We have added to the main text the number of back and forth unfolding/folding events average unfolding and folding times in the unbiased simulations. We also explicitly report the average unfolding and folding times. For Chignolin the mean unfolding time is 3.5 μs and the mean folding time is 0.3 μs. For TRP-cage, these quantities are 1.5 μs and 6.2 μs, respectively. We followed the dual-cutoff approach from Ref. [1] with a time window of 10 ns. We added the corresponding Matlab script to the paper's Github repository. We now report the simulations' temperature in the revised main text.

- Page 8, Figure 2: For the OneOPES simulation in Fig 2(c), is the simulation time shown on the x-axis, simulation time per replica or cumulative simulation time? This is an important distinction to have far comparison between the two simulation methods. Thus, I would ask the author to clarify this, and note this in the caption and text.

The simulation time that we report in that figure is the one local to replica 0. In the OneOPES approach that we use, a total of 8 replicas are run in parallel and attempt exchange of coordinates rather frequently. Computational cost is a delicate point when comparing single replica and multi-replica approaches, as one should take into account important considerations about parallelism. Running a single simulation 8 times longer is not really comparable in computational cost to running 8 shorter simulations in parallel, especially in an epoch like ours where parallel computing on GPUs is ubiquitous and extremely beneficial. In any case, there was no intention from us in the manuscript to explicitly compare the computational cost of the OPES and the OneOPES approaches. We have added a discussion on this point to page 8 of the revised manuscript.

- Page 9, Figure 3, for the 2D FESs in panels (c) and (f), the s^{HB} CV ranges from -60 to around 0, while the s^{SC} CV ranges from -15 to 20. For the s^{HB} CV, why is only showing negative values and why is the folded state around value of 0? Given the definition of the CV, I would think that the folded state would have higher positive values than exhibited in the 2D FESs.

The CV range that the reviewer observed is due to how the hydrogen bond (HB) features themselves are built. In each feature making up s^{HB}, the positive contribution is only given by one term that measures the presence of an HB contact. Because of the requirement of a continuous non-zero derivative for a CV to be used in enhanced sampling, its maximum value is less than 1. In the meantime, in the same feature there are a number of negative contributions that are due to terms that measure the presence of water or of non-native HB contacts with other protein atoms. Even if these terms are all short-range, their contribution is non-zero for the same considerations about the smooth derivatives above. Therefore, the noise added by the negative terms tends to add up and arbitrarily shift down the s^{HB} values towards the negative range, as the reviewer have observed. This has no effect on the quality of the simulations as the numerical value alone of a CV has no influence on an enhanced sampling simulation, only its derivative has.

To better visualise this point, in Fig. L-1, we show the violin plots of two hard HB and two soft HB features in chignolin, with the distribution for the folded state in blue and for the unfolded state in red. While the features correctly separate the states, their distributions tend to be shifted towards the negative range. In the revised manuscript, we have rephrased the part at page 7 where we had written that individual features become ≈ -1 in case of a non-native contact, by saying now that they go towards negative values.

To highlight the crucial role of the negative terms in the CVs, we performed a set of new single replica OPES simulations for Chignolin where we selectively turned off some or all of the negative terms. We have added their outcome to Fig. 2 in the main text and to Fig. S5 of the SI. The effect of removing the negative terms in the CVs is clearly unfavourable, with the simulations either taking longer to converge to the expected result (when turning off the contacts with water molecules) or even not converging within the given simulation time (when turning off non-native contacts with other protein atoms). This point is further discussed in the revised manuscript and in the SI.

- Page 14, Ref 4 and 5 are the same citation.

We have removed the duplicate citation.

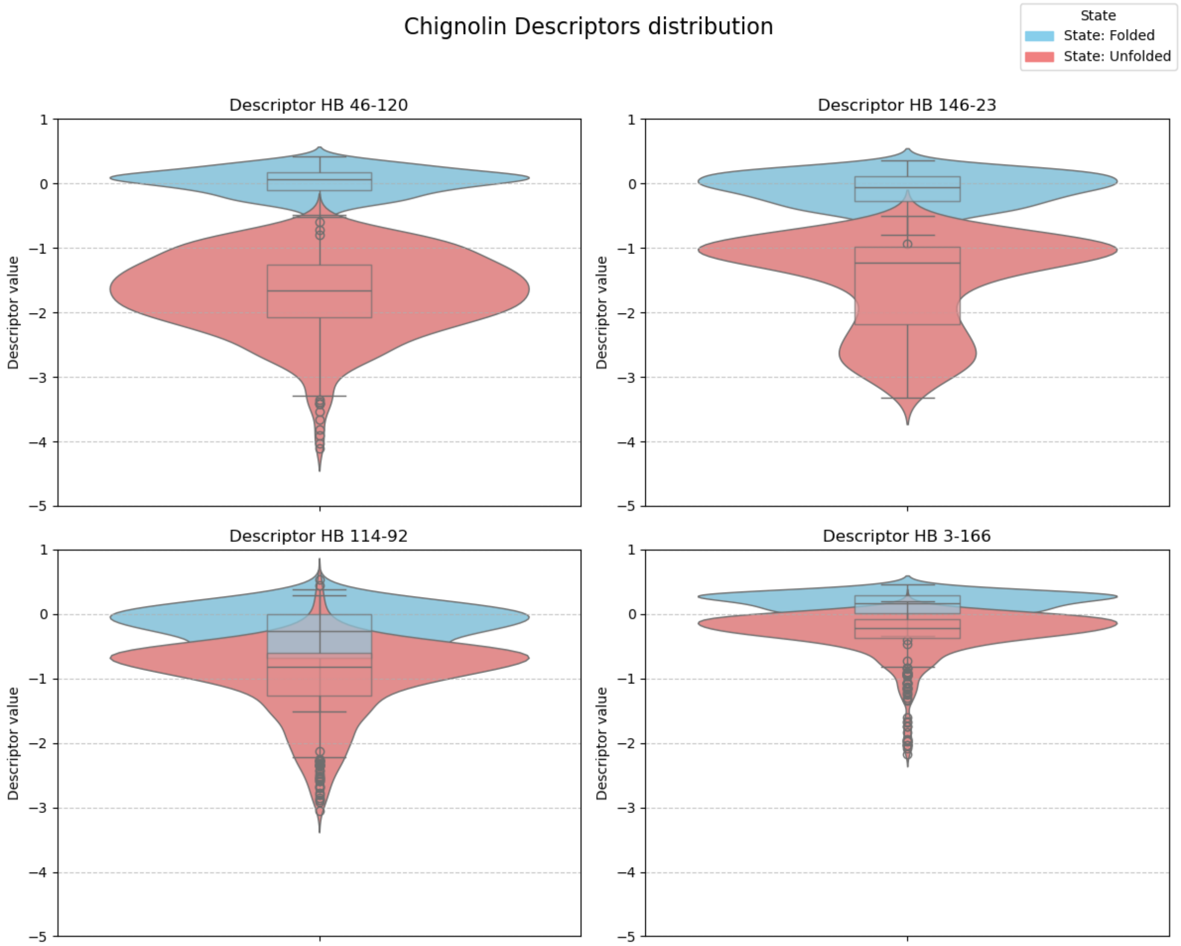

Figure L-1: Distribution of a selection of HB features in Chignolin over the folded state in blue and over the unfolded state in red.

References

[1] Kresten Lindorff-Larsen, Stefano Piana, Ron O Dror, and David E Shaw. How Fast-Folding Proteins Fold. *Science*, 334(6055):517–520, 10 2011.
